# Comparison of volumetric and 2D-based response methods in the PNOC-001 pediatric low-grade glioma clinical trial

**DOI:** 10.1093/noajnl/vdad172

**Published:** 2023-12-27

**Authors:** Marc von Reppert, Divya Ramakrishnan, Sarah C Brüningk, Fatima Memon, Sandra Abi Fadel, Nazanin Maleki, Ryan Bahar, Arman E Avesta, Leon Jekel, Matthew Sala, Jan Lost, Niklas Tillmanns, Manpreet Kaur, Sanjay Aneja, Anahita Fathi Kazerooni, Ali Nabavizadeh, MingDe Lin, Karl-Titus Hoffmann, Khaled Bousabarah, Kristin R Swanson, Daphne Haas-Kogan, Sabine Mueller, Mariam S Aboian

**Affiliations:** Department of Radiology and Biomedical Imaging, Yale School of Medicine, New Haven, Connecticut, USA; Department of Neuroradiology, Leipzig University Hospital, Leipzig, Germany; Department of Radiology and Biomedical Imaging, Yale School of Medicine, New Haven, Connecticut, USA; Department of Biosystems Science and Engineering, ETH Zürich, Basel, Switzerland; Swiss Institute for Bioinformatics (SIB), Lausanne, Switzerland; Department of Radiology and Biomedical Imaging, Yale School of Medicine, New Haven, Connecticut, USA; Department of Radiology and Biomedical Imaging, Yale School of Medicine, New Haven, Connecticut, USA; Department of Radiology and Biomedical Imaging, Yale School of Medicine, New Haven, Connecticut, USA; Department of Radiology and Biomedical Imaging, Yale School of Medicine, New Haven, Connecticut, USA; Department of Therapeutic Radiology, Yale School of Medicine, New Haven, Connecticut, USA; Center for Outcomes Research and Evaluation (CORE), Yale School of Medicine, New Haven, Connecticut, USA; Department of Neuroradiology, Harvard Medical School—Massachusetts General Hospital, Boston, Massachusetts, USA; Department of Radiology and Biomedical Imaging, Yale School of Medicine, New Haven, Connecticut, USA; University of Duisburg-Essen, Essen, Germany; Department of Radiology and Biomedical Imaging, Yale School of Medicine, New Haven, Connecticut, USA; Tulane School of Medicine, New Orleans, Louisiana, USA; Department of Radiology and Biomedical Imaging, Yale School of Medicine, New Haven, Connecticut, USA; Heinrich-Heine-Universität Düsseldorf, Düsseldorf, Germany; Department of Radiology and Biomedical Imaging, Yale School of Medicine, New Haven, Connecticut, USA; Heinrich-Heine-Universität Düsseldorf, Düsseldorf, Germany; Department of Radiology and Biomedical Imaging, Yale School of Medicine, New Haven, Connecticut, USA; Ludwig Maximilian University, Munich, Germany; Department of Therapeutic Radiology, Yale School of Medicine, New Haven, Connecticut, USA; Center for Outcomes Research and Evaluation (CORE), Yale School of Medicine, New Haven, Connecticut, USA; Center for Biomedical Image Computing and Analytics (CBICA), University of Pennsylvania, Philadelphia, Pennsylvania, USA; Center for Data-Driven Discovery in Biomedicine (D3b), Children’s Hospital of Philadelphia, Philadelphia, Pennsylvania, USA; Department of Radiology and Biomedical Imaging, Yale School of Medicine, New Haven, Connecticut, USA; Visage Imaging, Inc., San Diego, California, USA; Department of Neuroradiology, Leipzig University Hospital, Leipzig, Germany; Visage Imaging GmbH, Berlin, Germany; Mathematical Neuro-Oncology Lab, Department of Neurological Surgery, Mayo Clinic, Phoenix, Arizona, USA; Department of Radiation Oncology, Brigham and Women’s Hospital, Dana-Farber Cancer Institute, Boston Children’s Hospital, Harvard Medical School, Boston, Massachusetts, USA; Department of Neurology, Neurosurgery, and Pediatrics, UCSF, San Francisco, California, USA; Children’s University Hospital Zürich, Zürich, Switzerland; Department of Radiology and Biomedical Imaging, Yale School of Medicine, New Haven, Connecticut, USA

**Keywords:** Brain Tumor Reporting and Data System (BT-RADS), pediatric low-grade glioma (pLGG), Response Assessment in Neuro-Oncology (RANO), Response Assessment in Pediatric Neuro-Oncology (RAPNO), volumetrics

## Abstract

**Background:**

Although response in pediatric low-grade glioma (pLGG) includes volumetric assessment, more simplified 2D-based methods are often used in clinical trials. The study’s purpose was to compare volumetric to 2D methods.

**Methods:**

An expert neuroradiologist performed solid and whole tumor (including cyst and edema) volumetric measurements on MR images using a PACS-based manual segmentation tool in 43 pLGG participants (213 total follow-up images) from the Pacific Pediatric Neuro-Oncology Consortium (PNOC-001) trial. Classification based on changes in volumetric and 2D measurements of solid tumor were compared to neuroradiologist visual response assessment using the Brain Tumor Reporting and Data System (BT-RADS) criteria for a subset of 65 images using receiver operating characteristic (ROC) analysis. Longitudinal modeling of solid tumor volume was used to predict BT-RADS classification in 54 of the 65 images.

**Results:**

There was a significant difference in ROC area under the curve between 3D solid tumor volume and 2D area (0.96 vs 0.78, *P* = .005) and between 3D solid and 3D whole volume (0.96 vs 0.84, *P* = .006) when classifying BT-RADS progressive disease (PD). Thresholds of 15–25% increase in 3D solid tumor volume had an 80% sensitivity in classifying BT-RADS PD included in their 95% confidence intervals. The longitudinal model of solid volume response had a sensitivity of 82% and a positive predictive value of 67% for detecting BT-RADS PD.

**Conclusions:**

Volumetric analysis of solid tumor was significantly better than 2D measurements in classifying tumor progression as determined by BT-RADS criteria and will enable more comprehensive clinical management.

**Key Points**

- Solid tumor volume is more sensitive than 2D in classifying BT-RADS progression.
- Solid volume increase of 15–25% has optimal sensitivity for BT-RADS progression.

**Importance of the Study**

The Response Assessment in Pediatric Neuro-Oncology (RAPNO) criteria are based on bi-dimensional (2D) measurements. However, 2D measurements do not sufficiently capture the heterogeneous and diffuse nature of pediatric low-grade gliomas (pLGG). Volumetric measurement of pLGG may offer a more comprehensive approach to characterizing treatment response. Furthermore, there is a lack of empirical evidence for RAPNO-suggested volumetric thresholds, which are based on extrapolation of 2D thresholds to a perfect sphere. The present study demonstrates that volumetric analysis of solid tumor is significantly better than 2D measurements in classifying tumor progression as determined by BT-RADS criteria. In addition, a threshold of 15–25% provides better sensitivity for detecting PD than the current RAPNO volume-extrapolated threshold of 40%. In summary, volumetrics will enhance the detection of disease progression and lead to changes in clinical management.

Pediatric low-grade gliomas (pLGG) constitute approximately 30% of all central nervous system (CNS) neoplasms in children, making it the most common solid pediatric tumor.^1,2^ Ten-year overall survival of pLGG ranges from 85% to 95%, but morbidity associated with commonly used therapies, such as chemotherapy, targeted inhibitors, radiotherapy, and repeated surgeries, can lower quality of life and long-term outcomes.^3,4^ Accurate radiographic treatment response assessment in neuro-oncology is critical for guiding clinical management.

However, response assessment is challenging in patients with pLGG due to its heterogeneous imaging appearance and irregular borders. The Response Assessment in Pediatric Neuro-Oncology (RAPNO) working group^5^ formulated response criteria that consider the specific imaging characteristics of pLGG to help standardize the assessment: irregular and lobulated shapes, cystic components, highly variable patterns of enhancement, and lesion size. RAPNO criteria are based on 2D measurement of pLGG with emphasis on using the fluid-attenuated inversion recovery (FLAIR) sequence for measurements. In addition, RAPNO criteria provide guidelines for when to include cystic components of pLGG in tumor measurements. More recently, geometric extrapolation of 2D measurements to a spherical volume has been incorporated into RAPNO criteria. However, 2D methods are inherently variable, and this extends to its 3D extrapolation, along with added assumptions of uniform three-dimensional tumor growth. This may be why pLGG tumor response can differ between 2D and extrapolated 3D measurement methods.^6^ An actual volume from 3D segmentation is a more accurate representation of the true volumetric nature of the tumor.

The Brain Tumor Reporting and Data System (BT-RADS) criteria attempt to address the limitations in quantitative 2D criteria by providing a structured framework for neuroradiologists to classify tumor response based on multifactorial comparison of tumor on follow-up studies.^7^ The BT-RADS framework considers tumor enhancement, nonenhancing component, mass effect, and treatment-related phenomena when assigning tumor response scores, which are tied to definitive recommendations in clinical management.^7^ Although BT-RADS criteria may offer a more holistic approach to tumor response assessment, they are not currently widely used in neuro-oncology clinical trials, which still rely on quantitative measurements for tumor assessment. Thus, there is a need to determine whether 2D or volumetric-based quantitative response assessment more closely correlates with the BT-RADS framework since BT-RADS is more closely tied to clinical management recommendations.

The purpose of this study was to determine if volumetric analysis may provide a more accurate response assessment compared to 2D measurements in pLGG. We did this by evaluating volumetric and 2D response assessment methods in pLGG through a retrospective analysis of MRI data from the PNOC-001 phase II clinical trial of everolimus (NCT01734512) and comparing them to neuroradiologist visual assessment of tumor response based on BT-RADS criteria.^7^ We also longitudinally modeled posttreatment volumetric change to predict BT-RADS classification.

## Materials and Methods

### Participant Cohort

The PNOC-001 phase II trial of everolimus for recurrent or progressive pLGG (NCT01734512) included 65 participants aged 3–21 years enrolled from December 2012 to July 2019.^8,9^ Details on the mechanism of everolimus can be found in Supplementary Figure 1. Our volumetric analysis included 43 participants from the initial trial cohort of 65 (Table 1). Exclusion criteria included participants with only spinal cord tumors or those for whom a complete set of pretreatment and follow-up images could not be transferred from the clinical site into our research PACS for volumetric segmentation (Supplementary Figure 2). Informed consent was waived for our retrospective study as all imaging data was anonymized.

**Table 1.** Demographic and Baseline Imaging Characteristics for Participants

Age (y) at enrollment—median (min–max)	9 (3–19)
Sex—*N* (%)	
** **Male	24 (55.8)
** **Female	19 (44.2)
Diagnosis—*N* (%)	
** **Pilocytic astrocytoma	27 (62.8)
** **Astrocytoma (NOS)	8 (18.6)
** **Pilomyxoid astrocytoma	4 (9.3)
** **Pleomorphic xanthoastrocytoma	2 (4.7)
** **Other	2 (4.7)
Tumor location—*N* (%)	
** **Suprasellar/optic pathway	23 (53.5)
** **Supratentorial (other)	12 (27.9)
** **Posterior fossa	8 (18.6)
Magnetic field strength—*N* (%)	
** **3 T	23 (53.5)
** **1.5 T	20 (46.5)
Slice thickness (mm)—mean (SD), and spacing—mean (SD)	
** **FLAIR	3.6 (1.5), 4.4 (1.4)
** **T1CE	2.3 (1.7), 1.8 (2.3)
** **T2	4.0 (1.1), 4.5 (1.1)
Tumor volumetrics at baseline	
** **Presence of EC—*N* (%)	39 (90.7)
** **Presence of C—*N* (%)	34 (79.1)
** **WT volume (cm^3^)—mean (SD)	56.1 (82.8)
** **EC volume (cm^3^)—mean (SD)	14.9 (18.1)
** **C volume (cm^3^)—mean (SD)	7.8 (14.0)

SD = standard deviation, NOS = not otherwise specified, T1CE = T1-weighted contrast-enhanced, EC = enhancing core, C = cystic component, WT = whole tumor.

### Image Acquisition

Study participants were imaged at 18 participating institutions of the Pacific Pediatric Neuro-Oncology Consortium (PNOC). Brain MR images were acquired on 1.5 T and 3 T scanners using routine sequences (3 plane localizer, T2 weighted imaging, 3D FLAIR, and T1 weighted imaging without and with intravenous contrast (T1CE)). At the time of this analysis, 41 participants had pretreatment baseline images, and 2 participants, PNOC001-14 and PNOC001-62, had baseline images that occurred 1.7 and 0.7 months after treatment initiation, respectively. Participants had follow-up images taken at 2-month intervals. Two participants, PNOC001-35 and PNOC001-50, had 2 available pretreatment images, of which the earlier one was used as the baseline image. Overall, we analyzed a total of 213 postbaseline follow-up images across all 43 participants. The median number of postbaseline follow-up images per participant was 4 with a range of 1–10, while the median time from baseline for all follow-up images was 182 days with a range of 27–695.

### 2D Measurements

We then compared the original 2D measurements to volumetric measurements. A board-certified neuroradiologist (M.S.A.) performed the 2D measurements of solid tumor using Horos software on either FLAIR or T1CE based on which sequence and plane the solid tumor was best visualized and reliably measured on. These 2D measurements were part of the central imaging review for the original PNOC-001 trial. For tumors with cystic components, the cyst was included in the 2D measurements only if it was inseparable from the solid component. Cysts that occurred on the interface between solid tumor and healthy brain parenchyma were not included in the 2D measurements. Supplementary Table 1 summarizes how 2D measurements were performed for all participants.

### Volumetric Measurements

**Figure 1.**
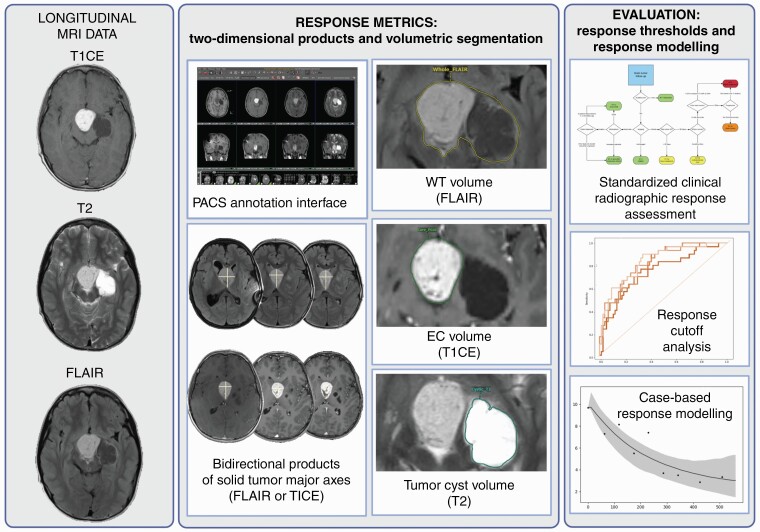
Volumetric annotation workflow: segmentations were performed in a PACS-integrated environment. ROC analysis was used to compare performance of 2D and volumetric percent change in classifying the response category assigned by neuroradiologist visual assessment using BT-RADS criteria.

To compare 2D with volumetric measurements, all included images were segmented using a previously described PACS-integrated annotation platform^10^ (AI Accelerator, Visage Imaging, Inc., San Diego, CA) (Figure 1). One medical student (M.v.R.) performed manual 3D segmentations of the cystic tumor component on T2, enhancing tumor core on T1CE, and whole tumor on FLAIR. The resection cavities, blood vessels, and dura were not included in the segmentations. All segmentations were reviewed and corrected by a board-certified neuroradiologist (M.S.A.).

### Response Assessment

The tumor 2D area was calculated by multiplying the orthogonal 2D measurements of solid lesion. Whole tumor volume was calculated from whole tumor segmentations on FLAIR, which included all cystic components and peritumoral edema. The sequence, either FLAIR or T1CE, which best showed a solid tumor on volumetrics as determined by neuroradiologist assessment was used to calculate solid tumor volume. Cystic components within the solid tumor region were triangulated with the T2 sequence, and their volume on T2 was subtracted from solid tumor volume measured on either FLAIR or T1CE. In studies with multiple solid lesions, the sum of the 2D area or 3D volume per lesion was analyzed on each follow-up image and compared to baseline to determine response. Supplementary Table 1 summarizes how solid volume was calculated for each participant.

### Comparison of 2D and Volumetrics in Response Assessment

Given the high survival rate for pLGG, it is challenging to use overall survival as an endpoint to validate 2D and volumetric response assessment. In practice, 2D-based radiographic progression-free survival along with clinical symptoms is often used to assess treatment response.^11^ We compared 2D and volumetric response assessment in a subset of 65 of 213 follow-up images to a clinical reference standard, the BT-RADS. The subset of 65 follow-up images was taken across all 43 participants and had a median follow-up time of 187 days (range = 65–695). Forty-three of the 65 follow-up images were considered “early time point” images and included 1 image per participant taken around the 6-month posttreatment follow-up. The remaining 22 of 65 images were considered “late time point” images and included 1 image per participant taken at the last available follow-up for the 22 participants who remained in the trial per protocol beyond the early time point.

The BT-RADS criteria are based on a multiplanar comparison of tumor on follow-up images and consider tumor enhancement, nonenhancing component, mass effect, and treatment-related phenomena to assign a score that ranges from BT-0 to BT-4.^7^ Each score represents a response category that is tied to a specific recommendation for clinical management and monitoring. Importantly, BT-RADS does not include quantitative measurements, unlike traditional 2D response criteria, and instead focuses on a holistic assessment of tumor change to classify response. BT-RADS provides the closest approximation of outcomes for these slow-growing indolent tumors. Following a simultaneous image presentation paradigm,^12^ 2 board-certified neuroradiologists (M.S.A. and F.M.), who were blinded to the outcome, independently assigned BT-RADS scores to the subset of 65 follow-up images compared to baseline with a third board-certified neuroradiologist resolving conflicts (S.A.F.). The baseline image was used for comparison since it is the recommended procedure for slow-growing lesions like pLGG^13^ and was also employed for comparison in the original trial.^9^

A weighted-kappa (*κ*_w_) statistic was calculated to measure inter-reader agreement for BT-RADS score assignments.

The final consensus BT-RADS score for each follow-up image was classified into the following 3 response categories: partial response (PR), stable disease (SD), and progressive disease (PD) to enable direct comparison to RAPNO classification (Table 2). We classified BT-RADS “1a” as PR since this score indicates imaging improvement most likely due to decreasing tumor burden and not treatment-related pseudoresponse. We classified BT-RADS “3c” and “4” as PD as these scores indicate imaging worsening most likely due to increasing tumor burden and not treatment-related pseudoprogression. Moreover, both categories suggest potential changes in clinical management. The remaining BT-RADS categories (ie, “1b,” “2,” “3a,” and “3b”) were classified as SD. Although “1b” indicates imaging improvement, it is likely due to treatment-related effects, and thus we did not include it as PR. Similarly, categories “3a” and “3b” indicate imaging worsening but not definitively due to an increase in tumor burden over treatment-related effects, and thus we did not include these scores as PD. In all analyses, RAPNO categories of minor response and partial response were grouped together as no such sub-differentiation was made for visual response assessment using BT-RADS.

**Table 2.** Criteria for Response Assessment

Response Assessment	Partial Response (PR)	Minor Response (MinR)	Stable Disease (SD)	Progressive Disease (PD)
Visual (BT-RADS)	BT-RADS 1a		BT-RADS1b, 2, 3a, 3b	BT-RADS 3c, 4
2D RANO (product of perpendicular diameters)	≥50% decrease	n/a	<25% increase, <50% decrease	≥25% increase or new lesion
2D RAPNO (product of perpendicular diameters)	≥50% decrease	25–50% decrease	<25% increase, <25% decrease	≥25% increase or new lesion
VOL RANO (volumetrics using RANO cutoffs)	≥50% decrease	n/a	<25% increase, <50% decrease	≥25% increase or new lesion
VOL RAPNO (volumetrics using RAPNO cutoffs)	≥50% decrease	25–50% decrease	<25% increase, <25% decrease	≥25% increase or new lesion
VOL-extrap. RANO (extrapolated for sphere)	≥65% decrease	n/a	<40% increase, <65% decrease	≥40% increase or new lesion
VOL-extrap. RAPNO (extrapolated for sphere)	≥65% decrease	35–65% decrease	<40% increase, <35% decrease	≥40% increase or new lesion

We compared response classification in the subset of 65 follow-up images using BT-RADS, 2D area applying standard RAPNO thresholds, and 3D solid volume applying volume-extrapolated RAPNO thresholds. The overall percent change from baseline in 2D and 3D measurements was used to compare response classification. We conducted 3 Friedman ANOVA tests to compare 2D area, 3D solid volume, and 3D whole volume percent change in each response group (PR, SD, and PD) for this same subset of 65 follow-up images. A one-way ANOVA was performed to compare total cystic volume percent change among follow-up images containing cystic components between the 3 BT-RADS groups.

Using the subset of 65 follow-up images, we also constructed empirical receiver operating characteristic (ROC) curves for each of the 3 measurement methods to classify BT-RADS determined PR and PD using the pROC package^14^ on RStudio version 2022.12.0 + 353. The area under the curve (AUC) was calculated with 95% confidence intervals obtained through 2,000 stratified bootstrap replicates. The DeLong test was used to determine whether there was a statistically significant difference in AUC between methods in classifying BT-RADS PR and PD. For 3D solid volume percent change, we determined the median sensitivity and specificity to detect BT-RADS PD with 95% confidence intervals obtained through 2,000 stratified bootstrap replicates for the thresholds from 15% to 40% (the current volume-extrapolated RAPNO threshold for PD) with 5% intervals and to detect BT-RADS PR for the thresholds from −15% to −65% (the current volume-extrapolated RAPNO threshold for PR). Since we assumed a measurement uncertainty of 10%, we considered thresholds starting at 15%. We determined the range of optimal thresholds as ones that included an 80% sensitivity within the 95% confidence interval for sensitivity.

### Modeling of Longitudinal Response

Finally, we made response predictions based on volumetric response trajectories. Traditionally, clinical assessment is based on a paired comparison of images acquired at 2 time points. This is agnostic of the underlying response and growth dynamics. Tumor growth curve analysis may provide additional insight to detect the onset of treatment resistance and regrowth early. Figure 3A shows schematic examples of typical tumor growth trajectories after treatment initiation characterized by an optional initial regression prior to regrowth. The time to reach the minimal tumor volume, *t*_Vmin_ is patient-specific and characteristic of response. Following previous work,^15,16^ we described this growth pattern using a mechanistic description of exponential tumor growth (at rate *λ*, constant across patients), treatment-induced shrinkage (*γ*_0_), and the onset of treatment resistance (*є*) (Figure 3A, see Section 3 in Supplementary Material for details). We fit the model to growth curves of the 3D solid tumor volume for each of the subset of 65 images with BT-RADS assessment if at least 3 prior images were available. We accounted for both fit uncertainty (using the Markov Chain Monte Carlo method implemented via the *emcee* algorithm) and volume measurement uncertainty (using bootstrapping, *N* = 200, assuming 10% uncertainty). We classify response on each image based on the evaluation time, *t*_eval_, relative to *t*_Vmin_, indicating a change in the net growth rate. Images with tumor volume that has clearly surpassed *V*_min_ (*t*_eval_*>* 75th percentile *t*_Vmin_) are labeled as PD. SD is classified in case of no significant change in any of the volume measurements from the pretreatment volume (scored as a deviation larger than the estimated measurement uncertainty) and PR otherwise. Hence, the model-based label accounts for the trajectory rather than a single endpoint. We further compare the obtained fit-parameter distributions between responding (*t*_Vmin_*≥*1 year) and resistant (*t*_Vmin_*<* 1 year) images using a Wilcoxon Rank Sum test. We compared model classification performance to BT-RADS assessment in the images with model fits.

## Results

### Inter-reader Agreement for Visual Response Assessment

Out of the subset of 65 follow-up images, 28 were classified as PD, 23 as SD, and 14 as PR by BT-RADS. Blinded BT-RADS assessment resulted in discordant reads in a total of 11 images from 9 different participants (observed agreements: 54, 83.1%), corresponding to a *κ*_w_ = 0.78, (*κ* = 0.73, 95% CI 0.586–0.874), which can be interpreted as “substantial agreement between readers,” nearing a perfect agreement. Discordant reads frequently occurred in the early time point images (~6 months follow-up, 8 of 11 images) when subtle changes in lesion size were classified as responding or progressing by 1 reader and as stable by the other. The inter-reader agreement was, therefore, higher (*κ*_w_ = 0.874, indicating “almost perfect agreement”) when considering only the last available follow-up images for all participants. In addition, 7 of the 9 participants with discordant inter-reader BT-RADS scores had tumors located in the suprasellar/optic pathway.

### Congruence Between 2D and Volumetric Methods in Response Assessment

**Figure 2.**
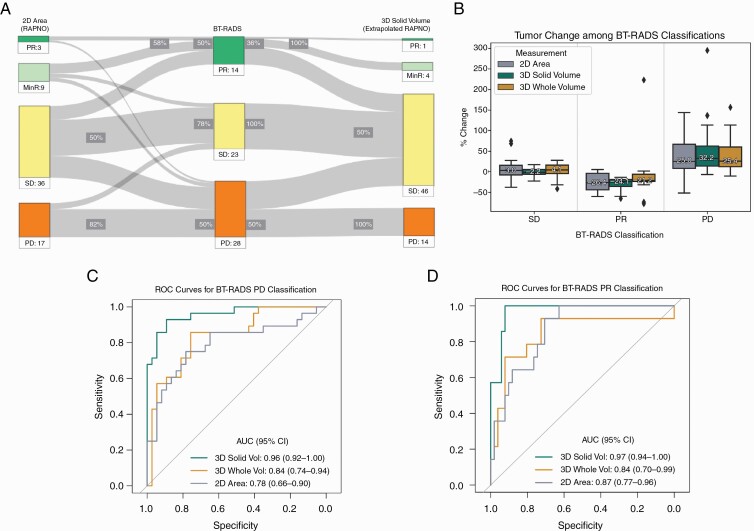
(A) Sankey diagram comparing 2D area to 3D solid volume using BT-RADS assessment as reference. Volumetric classifications employ the extrapolated 40% threshold for tumor progression. Sensitivities are shown as percentages next to the central BT-RADS column; specificities are shown closer to the 2D and 3D columns. (B) Comparison of percent tumor change between measurement methods within each group of images classified by BT-RADS. Comparison of ROC curves in classifying images as (C) BT-RADS PD and (D) BT-RADS PR.

First, we compared the percent change values in 2D and 3D solid volume of all 213 follow-up images from the 43 participants. Overall, 147 of 213 total follow-up images (69%) showed congruent increase or decrease in 2D and 3D solid volume percent change. Correlation between 2D and 3D solid tumor volume change for all 213 follow-up images is illustrated in Supplementary Figure 3. Next, we compared RAPNO to BT-RADS criteria for the subset of 65 follow-up images. In keeping with previous studies, we first compared the response between 2D area change with RAPNO thresholds (2D RAPNO) and 3D solid volume change with volume-extrapolated RAPNO thresholds (3D solid volume-extrapolated RAPNO), which have been derived in the literature by extrapolating 2D thresholds to a perfect spherical volume.^17^ The 2D RAPNO and 3D solid volume-extrapolated RAPNO classifications were compared to BT-RADS assessment in the subset of 65 follow-up images as shown in Figure 2A. Compared to BT-RADS, applying 2D RAPNO criteria yielded discordant classification in 26 of the subset of 65 follow-up images (40%), and applying 3D solid volume-extrapolated RAPNO criteria resulted in discordant classification of 23 of the subset of 65 follow-up images (35%). 2D RAPNO and 3D solid volume-extrapolated RAPNO both had a sensitivity of 50% for the detection of BT-RADS PD and 50% and 36%, respectively, for the detection of BT-RADS PR. The specificity of 2D RAPNO and 3D solid volume-extrapolated RAPNO for detecting BT-RADS PD was 82% and 100%, respectively, while it was 58% and 100%, respectively, for detecting BT-RADS PR.

For the 28 of 65 follow-up images classified as BT-RADS PD, the median percent change in 2D area was 25.0% (IQR: [7.7%, 66.7%]), 3D solid volume was 32.2% (IQR: [13.7%, 62.1%]), and 3D whole volume was 25.4% (IQR: [12.1%, 59.8%]). For the 14 of 65 follow-up images classified as BT-RADS PR, the median percent change in 2D area was −26.4% (IQR: [−44.3%, −3.8%]), 3D solid volume was −24.1% (IQR: [−36.0%, −19.1%]), and 3D whole volume was −21.2% (IQR: [−23.8%, −6.3%]). For the 23 of 65 follow-up images classified as BT-RADS SD, the median percent change in 2D area was 3.0% (IQR: [−8.1%, 15.6%]), 3D solid volume was −2.2% (IQR: [−6.3%, 5.2%]), and 3D whole volume was 4.1% (IQR: [−4.8%, 16.2%]). Friedman ANOVA revealed that there was no significant difference in median percent change between 2D area, 3D solid volume, and 3D whole volume in the BT-RADS PD (*P* = .24), PR (*P* = .40), or SD images (*P* = .55) in the subset of 65 follow-up images (Figure 2B). Of the 28 BT-RADS PD follow-up images, 18 had cystic components with the median percent change of total cystic volume being 2.2% (IQR: [0.8%, 19.1%]). Of the 14 follow-up images classified as BT-RADS PR, all had cystic components with the median percent change of total cystic volume being 0.6% (IQR: [0.3%, 3.8%]). Seventeen of the 23 BT-RADS SD follow-up images were cystic, and the median percent change of total cystic volume was 1.9% (IQR: [1.3%, 9.1%]). A one-way ANOVA revealed no significant difference in median percent change of total cystic volume between the 3 groups of BT-RADS follow-up images (*P* = .10).

#### Determining Optimal Method and Empirical Thresholds—

Using the subset of 65 follow-up images, the AUC for classifying BT-RADS PD from non-PD images was 0.78 (95% CI: [0.66, 0.90]) for 2D area, 0.84 (95% CI: [0.74, 0.94]) for 3D whole volume, and 0.96 (95% CI: [0.92, 1.00]) for 3D solid volume percent change (Figure 2C). There was a significant difference in AUC between 3D solid volume and 2D area percent change (*P* = .005) and between 3D solid and 3D whole volume percent change (*P* = .006). However, there was no significant difference in AUC between 3D whole volume and 2D area percent change (*P* = .51). Thresholds of 15%, 20%, and 25% increase in 3D solid volume included an 80% sensitivity in the 95% confidence interval for detecting BT-RADS PD (Table 3). The AUC for predicting BT-RADS PR from non-PR images was 0.87 (95% CI: [0.77, 0.96]) for 2D area, 0.84 (95% CI: [0.70, 0.99]) for 3D whole volume, and 0.97 (95% CI: [0.94, 1.00]) for 3D solid volume percent change (Figure 2D). There was a significant difference in AUC between 3D solid volume and 2D area percent change (*P* = .02). However, there was no significant difference in AUC between 3D solid and 3D whole volume percent change (*P* = .08) or between 3D whole volume and 2D area percent change (*P* = .81). Thresholds of −15% and −20% decrease in 3D solid volume included an 80% sensitivity in the 95% confidence interval for detecting BT-RADS PR (Table 3).

**Table 3.** Sensitivity and Specificity for 3D Solid Volume Percent Change Thresholds from 15% to 40% with 5% Intervals for Classifying BT-RADS PD and from −15% to −65% with 5% Intervals for Classifying BT-RADS PR Calculated from their Respective Empirical ROC Curves

BT-RADS Progressive Disease Classification		
Threshold	Median Sensitivity (95% CI)	Median Specificity (95% CI)
15%	0.75 (0.57–0.89)	0.97 (0.92–1.00)
20%	0.64 (0.46–0.82)	1.00 (1.00–1.00)
25%	0.64 (0.46–0.82)	1.00 (1.00–1.00)
30%	0.57 (0.39–0.75)	1.00 (1.00–1.00)
35%	0.50 (0.32–0.68)	1.00 (1.00–1.00)
40%	0.50 (0.32–0.68)	1.00 (1.00–1.00)

BT-RADS Partial Response Classification		
Threshold	Median Sensitivity (95% CI)	Median Specificity (95% CI)
−15%	0.86 (0.64–1.00)	0.94 (0.86–1.00)
−20%	0.64 (0.36–0.86)	0.94 (0.86–1.00)
−25%	0.50 (0.21–0.79)	1.00 (1.00–1.00)
−30%	0.43 (0.14–0.71)	1.00 (1.00–1.00)
−35%	0.36 (0.14–0.64)	1.00 (1.00–1.00)
−40%	0.21 (0.00–0.43)	1.00 (1.00–1.00)
−45%	0.14 (0.00–0.36)	1.00 (1.00–1.00)
−50%	0.14 (0.00–0.36)	1.00 (1.00–1.00)
−55%	0.14 (0.00–0.36)	1.00 (1.00–1.00)
−60%	0.14 (0.00–0.36)	1.00 (1.00–1.00)
−65%	0.07 (0.00–0.21)	1.00 (1.00–1.00)

### Tumor Response Modeling

**Figure 3.**
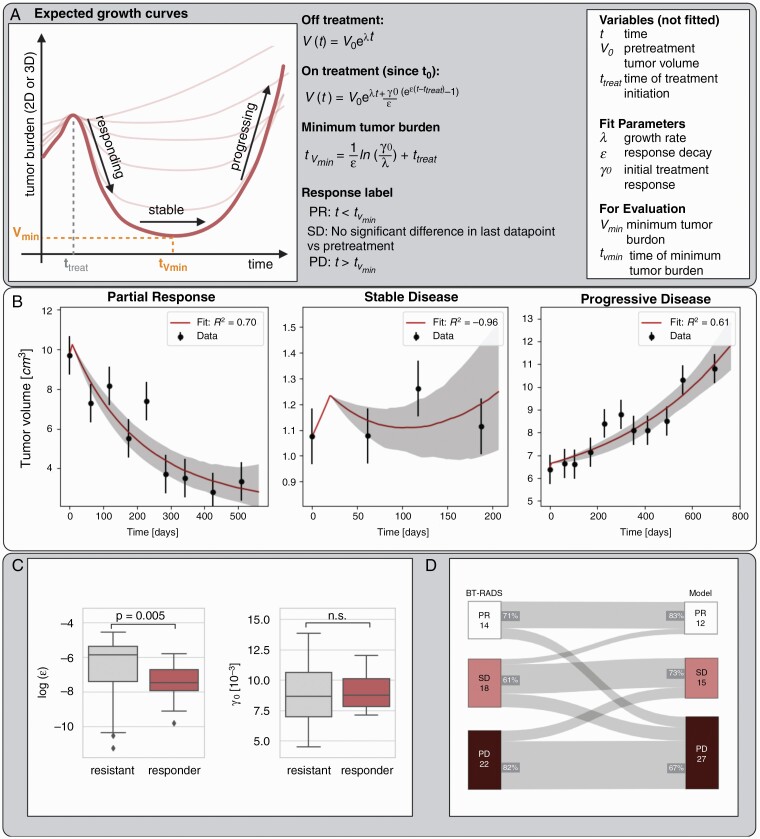
(A) Schematic overview of expected tumor growth curves with a mechanistic description through an ordinary-differential-equation-based model. (B) Tumor growth curves for selected images classified as PR, SD, or PD by the model. Shaded areas correspond to 95% prediction bounds. (C) Box plots visualizing the fit-parameter ranges between responding and resistant images. ^**^P-value < .01. (D) Sankey plot illustrating the agreement between BT-RADS and model-based classification of 54 images.

Fifty-four out of the subset of 65 follow-up images with BT-RADS assessment had at least 3 prior images to allow for model fitting. Thus, in the following analyses, only these 54 images were included. Figure 3B shows representative examples of participant data with model fits for PR, SD, and PD assessed at the last data point (Supplementary Figures 5–8 for all fit results). We analyzed the difference in fit parameters between responding and resistant images in Figure 3C. Responders were fitted with significantly lower ϵ (*P* = .005) but no difference in the initial treatment response (*γ*_0_) was observed. Figure 3D shows the Sankey diagram illustrating the agreement between the BT-RADS and model-assigned image assessments. We observed mainly transitions between BT- RADS SD and PD, however, changes in response from BT-RADS PD to SD and PR were also observed. This is due to the different evaluation endpoints, where modeling considers the full trajectory rather than a single evaluation endpoint. One participant (PNOC001-22) displayed significant contouring variation leading to discrepant assessments. The model had an 82% sensitivity for detecting BT-RADS PD and 71% sensitivity for BT-RADS PR. The positive predictive value of the model for detecting BT-RADS PD was 67% and 83% for BT-RADS PR. The negative predictive value of the model for detecting BT-RADS PD was 73% and 100% for BT-RADS PR.

## Discussion

This study investigates how volumetric criteria can be incorporated into radiographic response assessment in pLGG clinical trials. As the response assessment for these tumors relies heavily on radiographic evaluations, we used BT-RADS as a reference. This study extends prior published literature: (1) we show that solid volume measures are more accurate than bidirectional measurements in capturing tumor response for pLGG based on neuroradiologist visual assessment; (2) we propose new empirically derived solid volume thresholds for classifying tumor response and progression; and (3) we use the trajectory of longitudinal solid volume measurements to make model-based predictions that are highly consistent with BT-RADS visual assessment.

We first show that solid volume measurements are more accurate than bidirectional measures in classifying tumor progression based on BT-RADS in pLGG. While there was no difference between 2D, solid, or whole volume percent change among images classified in BT-RADS PD, SD, or PR response groups, change in 3D solid volume was significantly better than 2D area at classifying BT-RADS PD and PR in the subset of 65 follow-up images. This may be explained by the fact that solid volume was measured on the sequence that best showed the entirety of the tumor volume on all planes unlike 2D measurements, which are limited to a single plane. If the tumor has variable or irregular growth patterns, solid volume may better capture these changes. 3D solid volume was also significantly better than 3D whole volume in classifying BT-RADS PD. The mean total cystic volume on baseline images comprised about 14% of the mean whole tumor volume with little change in the cystic volume in 32 of 43 trial participants.

Moreover, there was no significant difference in total cystic volume change between images classified as BT-RADS PR, SD, or PD. Together, these findings suggest that cystic changes may not have significantly influenced BT-RADS assessment in our cohort which can explain why their exclusion from solid volume measurements led to better BT-RADS PD classification than whole volume. Nevertheless, it is important to note that tumor cysts, which can lead to mass effect or compression of solid tumor,^5^ may influence response assessment, especially since BT-RADS includes evaluation of tumor mass effect in its criteria. Although this did not appear to be the case in our cohort, further research is needed to understand the specific cases and types of tumor cysts (eg, intratumoral vs reactive) that affect response assessment. Steroid administration can affect peritumoral edema,^18^ which was included in whole tumor segmentations. Since peritumoral edema can be difficult to differentiate from tumor invasion, we included it in our segmentations. However, peritumoral edema is less extensive in pediatric brain tumors.^19^ No images were classified as BT-RADS “1b” (ie, improvement likely due to treatment effects). So, it is more likely that whole volume and 2D performed similarly in BT-RADS classification due to inclusion of cyst as opposed to peritumoral edema.

Other studies have also suggested the superiority of volumetrics over bidirectional measurements in predicting tumor response. Ellingson et al.^20^ reported higher inter-reader agreement for volumetric compared to bidirectional measurements in adult low-grade gliomas. For pLGG, there are many sources of measurement variability, including acquisition parameters, shape across lesions, and head position within the scanner.^21–24^ D’Arco et al. demonstrated a 20% discordance in response assessment between bidirectional measurements and manual volumetric segmentation.^6^

Our study is the first to use BT-RADS visual assessment as a reference for comparison of volumetric and 2D measurement methods. This is relevant to our participant cohort since subtle radiological changes detected by BT-RADS can inform disease progression or response. We further propose a range of empirically defined thresholds to classify progressive disease and partial response in future volumetric studies. The current response cutoffs for bidirectional measures are rooted in the MacDonald criteria for GBM,^25^ that stipulate a 50% decrease for PR and 25% increase for PD to reduce the margin of error. For volumetric studies, these thresholds were extrapolated to 65% and 40%, respectively, following Chappell et al.^17^ assuming extrapolation of 2D measurements to a perfect spherical tumor shape. Since pLGGs are heterogeneous entities with irregular borders, we sought to define a new empirical threshold for PD and PR based on solid volume 3D segmentations. We found that a threshold of 40% in 3D solid volume increase resulted in a low median sensitivity of 50% with a 95% confidence interval of 32–68%. While our empirical threshold calculations are limited by sample size, they suggest a lower threshold in the range of 15–25% to detect PD with higher sensitivity.

Finally, we use longitudinal volumetric measurements to model tumor response trajectories. Pairwise comparative analysis of images is intrinsically agnostic of the underlying growth dynamics. The same tumor imaged during a period of regression should be interpreted differently when acquired in progressive growth phase. By means of mechanistic modeling, we provided such a dynamic context. By building this description on a few participant-specific parameters in combination with visualization of fit confidence, we provide a more detailed picture of the current state of a participant in context. The derived model-assigned response assessments further showed good agreement with BT-RADS. Where BT-RADS PD was not detected by the model (Supplementary Figure 5), decisions were indeed debatable and often due to small volumetric growth (below the fixed 10% uncertainty). Importantly, modeling identified cases that, despite no obvious volumetric increase at assessment, should be considered PD given the context of regrowth following short-term tumor shrinkage. Other studies have recently acknowledged the importance of longitudinal growth response in addition to paired image comparison, citing increased reproducibility and decision support for less experienced investigators as potential benefits.^26^

In an editorial on the most recent LGG volumetric study,^20^ Gerstner pointed out that the main limitation to incorporating volumetrics into clinical trials and practice includes time and cost.^27^ Our study used a PACS-based segmentation tool to perform volumetric analysis in a timely manner. This approach allowed for segmentations to be performed directly on the DICOM images within the PACS interface. Although the volumetric tool enabled extrapolation of segmentations, substantial manual adjustment was still necessary. Development of auto-segmentation algorithms for pLGG may facilitate volumetrics in practice. Although glioma auto-segmentation algorithms are more developed in adult populations given the greater availability of public datasets,^28^ recent studies indicate that translation of these algorithms to pediatric populations is promising.^29,30^ Some debate remains as to which tumor components should be segmented and assessed; for example, spontaneously varying components of enhancement in pLGG warrant further study.^31^ However, workflows that incorporate specific tumor sub-components as outlined by RAPNO recommendations in their segmentation paradigm are feasible.^32^

There are limitations to our study. First, despite our use of neuroradiologist visual assessment as a reference standard, we were not able to correlate volumetric measurements with functional outcomes, which is often a significant challenge with low-grade gliomas.^6,20^ Indeed, volumetrics has shown mixed evidence in the prediction of overall survival in glioblastomas. While 1 study showed that volumetrics did not improve prognosis over 2D measurements when performed within the first 12 weeks posttreatment,^33^ another study showed that the necrosis to tumor volume ratio was an important biomarker of prognosis in glioblastomas.^34^ Second, because the trial started enrollment more than a decade ago, imaging parameters do not necessarily conform to recommendations stated in the recently published RAPNO guidelines.^5^ This resulted in differences in acquisition parameters such as slice thickness or gap, which may affect volume calculation.^22^ Furthermore, it must be noted that some patients were measured on FLAIR sequences and others on T1CE, considering which sequences visualized solid tumor components best. We may thus incur an inconsistency in our approach. However, this is in line with clinical practice, where the sequence that best visualizes the tumor is used to determine tumor response. Measurements were performed by 1 neuroradiologist, limiting our ability to assess how inter-reader variability can impact results. In our cohort, solid tumor components were often well-circumscribed, which would contribute to better inter- and intra-reader agreement overall.^35^ It is important to note that we compared 3 measurement methods in isolation to predict BT-RADS classification when, in fact, a combination of tumor components, including solid and cystic, may influence response classification. However, due to time limitations in clinical practice, we sought to evaluate the best individual classifier for tumor response. Furthermore, our analysis included follow-up images with a median of 6 months after treatment initiation. Volumetrics may perform better than 2D at earlier time points, warranting further research into its performance at later time points posttreatment.

Future work should explore the feasibility of auto-segmentation and longitudinal posttreatment segmentation in pediatric cohorts, like recent work presented for adult populations.^36^ Attention should be given to incorporating methods of uncertainty quantification to estimate the reliability of automatically generated segmentations. We anticipate that the combination of both methods will decrease segmentation variability rendering volumetric criteria even more useful. Beyond the PACS-integrated tools for annotation and volumetric response criteria presented, PACS-workflow incorporation of these additional methods can substantially enhance clinical trial practice and better assess patient outcomes.

## Supplementary Material

vdad172_suppl_Supplementary_Figures_S1-S8_Tables_S1-S2
