# Supplementary material for: Comparison of volumetric and 2D-based response methods in the PNOC-001 pediatric low-grade glioma clinical trial
Source: Neurooncol Adv. 2023 Dec 27;6(1):vdad172. doi: 10.1093/noajnl/vdad172 (PMC10785766; doi:10.1093/noajnl/vdad172)
Supplement: vdad172_suppl_Supplementary_Figures_S1-S8_Tables_S1-S2 [file vdad172_suppl_supplementary_figures_s1-s8_tables_s1-s2.docx]

# 1 Clinical Trial Background

The last decade saw abundant progress in the effort to elucidate molecular drivers for pediatric low-grade gliomas and initiation of many clinical trials for therapies targeting players involved in Ras-Raf-MAP kinase and PI3K-AKT-mTOR signaling. The largest study to date on molecular pathogenesis and clinical outcomes of pediatric low-grade gliomas showed that the Ras-Raf-MAP kinase pathway is implicated in the vast majority of cases, with *KIAA1549*-*BRAF* fusion, *NF1* and *BRAF* ^V600E^ mutation accounting for nearly two thirds of all pLGG [[1](#_bookmark2)].

The PI3K-AKT-mTOR pathway is another interesting target for molecular therapies as it integrates a wide range of intracellular and extracellular signals to promote protein synthesis, cell survival, and cell metabolism, thus making it a key pathway in a range of cancers [[2](#_bookmark3), [3](#_bookmark4)]. Furthermore, both pathways exhibit significant crosstalk and interdependency, which led to pLGG being coined a “RAS/RAF/mTOR pathway disease” [[4](#_bookmark5), [5](#_bookmark6)]. For treatment of pLGG, previous trials have evaluated the efficacy of everolimus, which is a rapamycin analog and direct mTOR inhibitor. It received FDA approval for treatment of renal cell carcinoma in 2009 [[6](#_bookmark7)] and has been evaluated in several phase II studies for treatment of pLGG, where overall good tolerability and effectiveness for lesion stabilization was shown [[5](#_bookmark6), [7](#_bookmark8), [8](#_bookmark9)]. Its mechanism for inhibition of mTOR is depicted in **Figure** [**S1**](#_bookmark0).


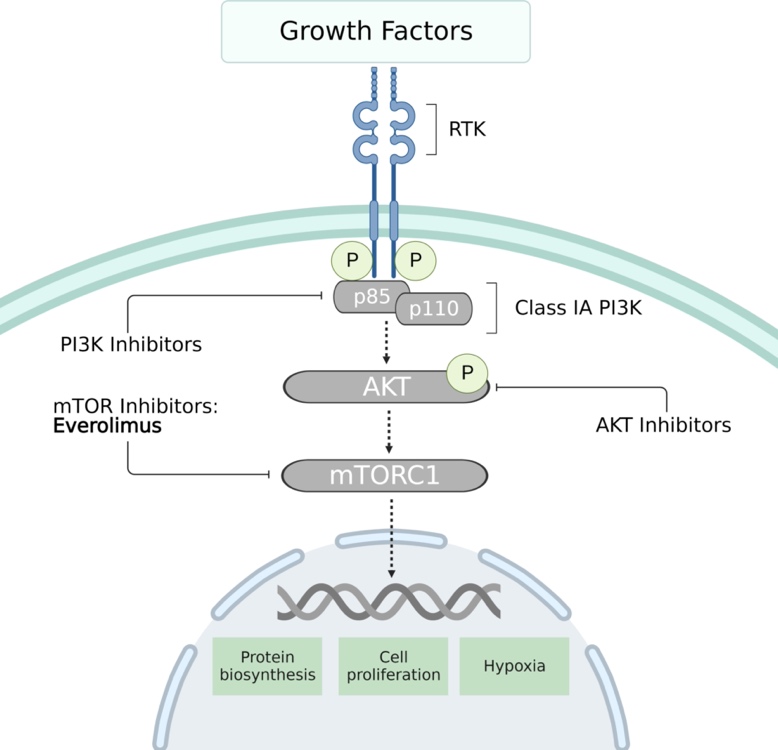


Created with BioRender.com

**Figure S1.** Everolimus is a direct mTOR inhibitor and has a more selective affinity for the mTORC1 protein complex than for the mTORC2 complex.

2 History of Clinical Trial Response Metrics

In pediatric low-grade gliomas, classification of anticancer treatment response heavily relies on neuroimaging since development of new clinical outcomes may become apparent only years after progression is seen radiographically. However, until recently, no such standard was in place for pediatric brain tumors, causing clinical trials to commonly resort to the RANO criteria [[9](#_bookmark10)] or a modified version thereof, which also includes minor response as a category [[10](#_bookmark11)].

Compared to the previous two-dimensional MacDonald [[11](#_bookmark12)] and unidimensional RECIST criteria [[12](#_bookmark13)] which captured only the enhancing tumor portion, incorporation of fluid-attenuated inversion recovery (FLAIR) in RANO criteria provided a significant improvement, in particular for response assessment of low-grade gliomas. Even so, there remained evident shortcomings in trials for pediatric brain tumors due to its design for and validation on adult-type tumors only [[13](#_bookmark14)]. This sparked the formation of the Response Assessment in Pediatric Neuro-Oncology (RAPNO) working group and an initial formulation of standardized recommendations for clinical trials of anticancer drugs in pLGG [[14](#_bookmark15)].

Among other items, the updates included an increased focus on functional patient outcomes, recommendations for a standardized imaging protocol across participating institutions as well as a more prominent role for FLAIR/T2-weighted imaging and incorporation of tumor cysts in response assessment. Radiographic response assessment builds on the previously defined RANO response categories for two-dimensional measurement (complete response (CR), partial response (PR), stable disease (SD) and progressive disease (PD)), with the additional provision of applicability in linear measurements in three perpendicular planes. Minor response and major response, characterized respectively by a 25-49% reduction in target lesion size and a 75% or greater reduction in size, paired with clinical stability or improvement of symptoms, were also included in the recommendations.

Table S1 summarizes trial details for the participants analyzed in this retrospective study as well as the way 2D measurements (i.e., sequence and plane) and measurement of solid tumor volume was performed.

| **Participant** | **Baseline Image** | **Details** |
| --- | --- | --- |
| PNOC001-11 | 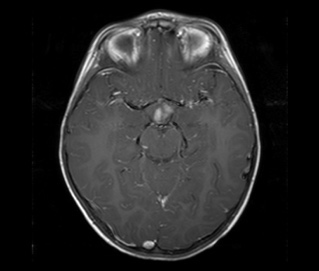 | **Age:** 5  **Sex:** male  **Diagnosis:** pilocytic astrocytoma  **Tumor location:** suprasellar/optic pathway  **Baseline total cystic volume (cm^3^):** 0.1  **Number of follow-up images:** 5  **Treatment duration (months):** 10  **Early timepoint from baseline image (months):** 6  **Late timepoint from baseline image (months):** 10  **2D measurement:** T1CE sagittal  **Solid volume measurement:** FLAIR minus total cyst |
| PNOC001-12 | 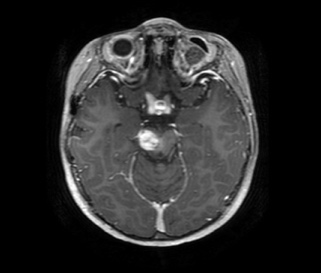 | **Age:** 5  **Sex:** female  **Diagnosis:** pilocytic astrocytoma  **Tumor location:** posterior fossa  **Baseline total cystic volume (cm^3^):** 0.2 (cyst appears on 1st follow-up)  **Number of follow-up images:** 10  **Treatment duration (months):** 22  **Early timepoint from baseline image (months):** 6  **Late timepoint from baseline image (months):** 23  **2D measurement:** T1CE axial  **Solid volume measurement:** FLAIR minus total cyst |
| PNOC001-14 | 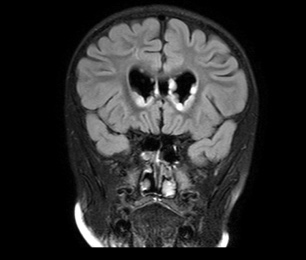 | **Age:** 7  **Sex:** male  **Diagnosis:** pilocytic astrocytoma  **Tumor location:** supratentorial  **Baseline total cystic volume (cm^3^):** N/A  **Number of follow-up images:** 8  **Treatment duration (months):** 22  **Early timepoint from baseline image (months):** 6  **Late timepoint from baseline image (months):** 21  **2D measurement:** T1CE coronal  **Solid volume measurement:** FLAIR |
| PNOC001-15 | 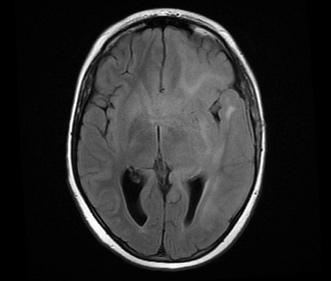 | **Age:** 12  **Sex:** female  **Diagnosis:** astrocytoma  **Tumor location:** supratentorial  **Baseline total cystic volume (cm^3^):** N/A  **Number of follow-up images:** 3  **Treatment duration (months):** 4.5  **Early timepoint from baseline image (months):** 4.5  **Late timepoint from baseline image (months):** N/A  **2D measurement:** FLAIR axial  **Solid volume measurement:** FLAIR |
| PNOC001-16 | 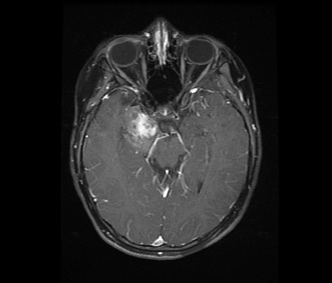 | **Age:** 6  **Sex:** male  **Diagnosis:** pleomorphic xanthoastrocytoma  **Tumor location:** supratentorial  **Baseline total cystic volume (cm^3^):** 1.4  **Number of follow-up images:** 2  **Treatment duration (months):** 2.5  **Early timepoint from baseline image (months):** 4  **Late timepoint from baseline image (months):** N/A  **2D measurement:** T1CE coronal  **Solid volume measurement:** FLAIR minus total cyst |
| PNOC001-17 | 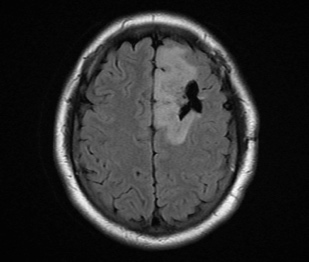 | **Age:** 19  **Sex:** male  **Diagnosis:** astrocytoma  **Tumor location:** supratentorial  **Baseline total cystic volume (cm^3^):** N/A  **Number of follow-up images:** 2  **Treatment duration (months):** 2  **Early timepoint from baseline image (months):** 5  **Late timepoint from baseline image (months):** N/A  **2D measurement:** FLAIR axial  **Solid volume measurement:** FLAIR |
| PNOC001-18 | 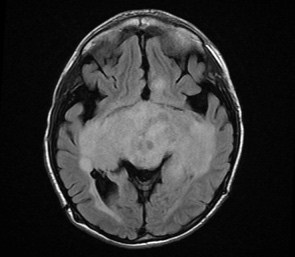 | **Age:** 3  **Sex:** male  **Diagnosis:** pilocytic astrocytoma  **Tumor location:** supratentorial  **Baseline total cystic volume (cm^3^):** N/A  **Number of follow-up images:** 3  **Treatment duration (months):** 9  **Early timepoint from baseline image (months):** 6  **Late timepoint from baseline image (months):** 8  **2D measurement:** T1CE axial  **Solid volume measurement:** FLAIR |
| PNOC001-21 | 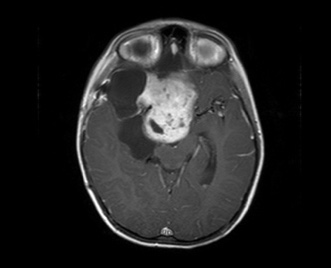 | **Age:** 4  **Sex:** female  **Diagnosis:** pilocytic astrocytoma  **Tumor location:** suprasellar/optic pathway  **Baseline total cystic volume (cm^3^):** 81  **Number of follow-up images:** 3  **Treatment duration (months):** 6  **Early timepoint from baseline image (months):** 6  **Late timepoint from baseline image (months):** N/A  **2D measurement:** T1CE axial  **Solid volume measurement:** T1CE |
| PNOC001-22 | 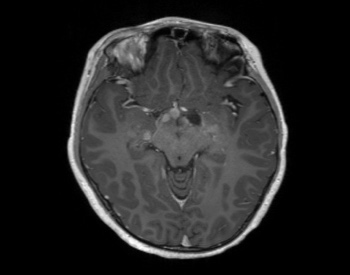 | **Age:** 8  **Sex:** male  **Diagnosis:** pilocytic astrocytoma  **Tumor location:** suprasellar/optic pathway  **Baseline total cystic volume (cm^3^):** 0.5  **Number of follow-up images:** 8  **Treatment duration (months):** 16  **Early timepoint from baseline image (months):** 7  **Late timepoint from baseline image (months):** 14  **2D measurement:** T1CE sagittal  **Solid volume measurement:** FLAIR minus total cyst |
| PNOC001-23 | 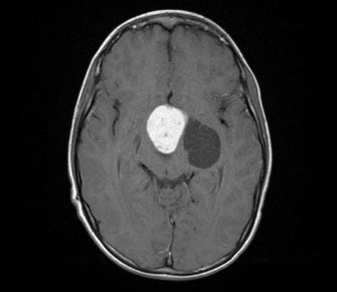 | **Age:** 9  **Sex:** male  **Diagnosis:** pilocytic astrocytoma  **Tumor location:** suprasellar/optic pathway  **Baseline total cystic volume (cm^3^):** 18  **Number of follow-up images:** 7  **Treatment duration (months):** 18.5  **Early timepoint from baseline image (months):** 6  **Late timepoint from baseline image (months):** 17  **2D measurement:** T1CE sagittal  **Solid volume measurement:** T1CE minus cyst inside enhancing volume |

| PNOC001-24 | 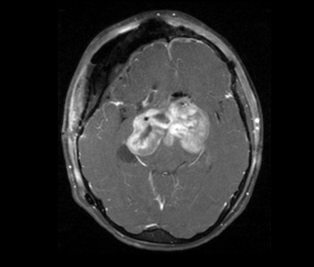 | **Age:** 12  **Sex:** male  **Diagnosis:** pilocytic astrocytoma  **Tumor location:** suprasellar/optic pathway  **Baseline total cystic volume (cm^3^):** 10  **Number of follow-up images:** 3  **Treatment duration (months):** 4  **Early timepoint from baseline image (months):** 5  **Late timepoint from baseline image (months):** N/A  **2D measurement:** T1CE coronal  **Solid volume measurement:** T1CE minus cyst within enhancing volume |
| --- | --- | --- |
| PNOC001-26 | 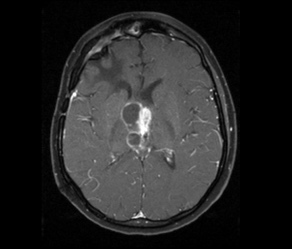 | **Age:** 13  **Sex:** female  **Diagnosis:** pilocytic astrocytoma  **Tumor location:** supratentorial  **Baseline total cystic volume (cm^3^):** 11.3  **Number of follow-up images:** 2  **Treatment duration (months):** 4  **Early timepoint from baseline image (months):** 4  **Late timepoint from baseline image (months):** N/A  **2D measurement:** T1CE coronal  **Solid volume measurement:** T1CE |
| PNOC001-27 | 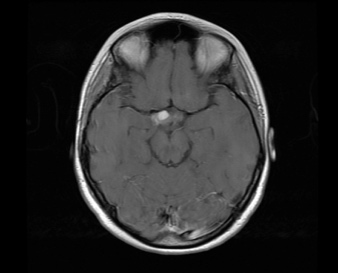 | **Age:** 15  **Sex:** female  **Diagnosis:** pilocytic astrocytoma  **Tumor location:** suprasellar/optic pathway  **Baseline total cystic volume (cm^3^):** 0.4  **Number of follow-up images:** 8  **Treatment duration (months):** 22  **Early timepoint from baseline image (months):** 6  **Late timepoint from baseline image (months):** 23  **2D measurement:** T1CE coronal  **Solid volume measurement:** FLAIR minus total cyst |
| PNOC001-29 | 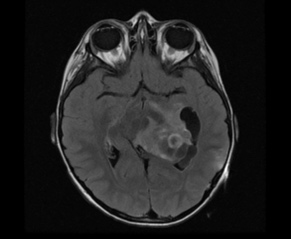 | **Age:** 9  **Sex:** male  **Diagnosis:** astrocytoma  **Tumor location:** supratentorial  **Baseline total cystic volume (cm^3^):** 18.5  **Number of follow-up images:** 2  **Treatment duration (months):** 4  **Early timepoint from baseline image (months):** 4  **Late timepoint from baseline image (months):** N/A  **2D measurement:** T2 axial  **Solid volume measurement:** FLAIR minus total cyst |
| PNOC001-30 | 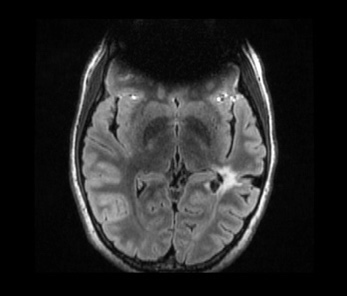 | **Age:** 14  **Sex:** female  **Diagnosis:** oligodendroglioma  **Tumor location:** supratentorial  **Baseline total cystic volume (cm^3^):** N/A  **Number of follow-up images:** 5  **Treatment duration (months):** 23  **Early timepoint from baseline image (months):** 6  **Late timepoint from baseline image (months):** 11  **2D measurement:** FLAIR axial  **Solid volume measurement:** FLAIR |

| PNOC001-32 | 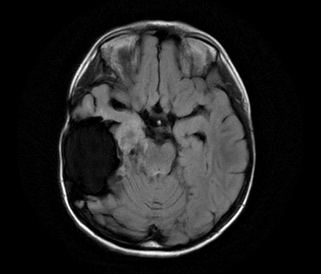 | **Age:** 12  **Sex:** female  **Diagnosis:** pleomorphic xanthoastrocytoma  **Tumor location:** supratentorial  **Baseline total cystic volume (cm^3^):** 7.1  **Number of follow-up images:** 2  **Treatment duration (months):** 8  **Early timepoint from baseline image (months):** 4  **Late timepoint from baseline image (months):** N/A  **2D measurement:** T1CE axial  **Solid volume measurement:** T1CE |
| --- | --- | --- |
| PNOC001-33 | 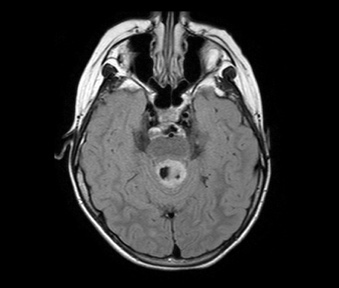 | **Age:** 6  **Sex:** female  **Diagnosis:** pilocytic astrocytoma  **Tumor location:** posterior fossa  **Baseline total cystic volume (cm^3^):** N/A  **Number of follow-up images:** 10  **Treatment duration (months):** 22  **Early timepoint from baseline image (months):** 6  **Late timepoint from baseline image (months):** 23  **2D measurement:** FLAIR axial  **Solid volume measurement:** FLAIR |
| PNOC001-35 | 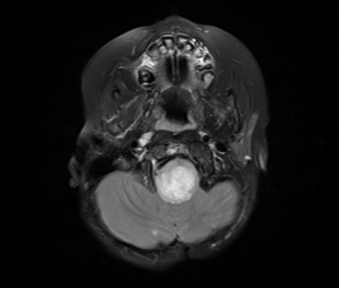 | **Age:** 3  **Sex:** female  **Diagnosis:** pilocytic astrocytoma  **Tumor location:** posterior fossa  **Baseline total cystic volume (cm^3^):** 0.15  **Number of follow-up images:** 3  **Treatment duration (months):** 4  **Early timepoint from baseline image (months):** 5  **Late timepoint from baseline image (months):** N/A  **2D measurement:** T2 sagittal  **Solid volume measurement:** FLAIR minus total cyst |
| PNOC001-36 | 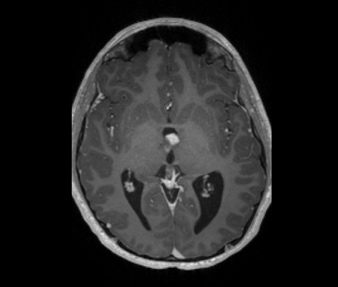 | **Age:** 15  **Sex:** male  **Diagnosis:** pilocytic astrocytoma  **Tumor location:** suprasellar/optic pathway  **Baseline total cystic volume (cm^3^):** N/A  **Number of follow-up images:** 10  **Treatment duration (months):** 22  **Early timepoint from baseline image (months):** 6  **Late timepoint from baseline image (months):** 23  **2D measurement:** T1CE sagittal  **Solid volume measurement:** FLAIR |
| PNOC001-37 | 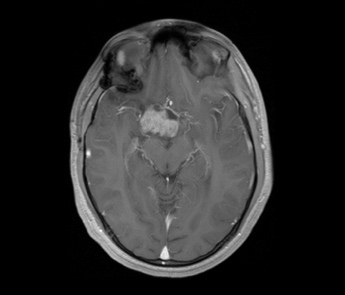 | **Age:** 13  **Sex:** male  **Diagnosis:** pilocytic astrocytoma  **Tumor location:** suprasellar/optic pathway  **Baseline total cystic volume (cm^3^):** 2.4  **Number of follow-up images:** 9  **Treatment duration (months):** 20  **Early timepoint from baseline image (months):** 6  **Late timepoint from baseline image (months):** 20  **2D measurement:** T1CE sagittal  **Solid volume measurement:** T1CE |

| PNOC001-38 | 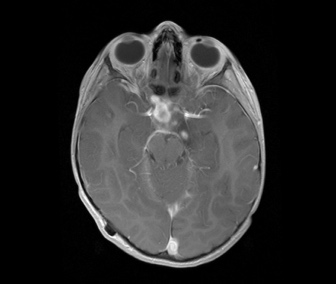 | **Age:** 7  **Sex:** female  **Diagnosis:** astrocytoma  **Tumor location:** suprasellar/optic pathway  **Baseline total cystic volume (cm^3^):** 9.1  **Number of follow-up images:** 2  **Treatment duration (months):** 4  **Early timepoint from baseline image (months):** 4  **Late timepoint from baseline image (months):** N/A  **2D measurement:** T1CE sagittal  **Solid volume measurement:** FLAIR minus total cyst |
| --- | --- | --- |
| PNOC001-39 | 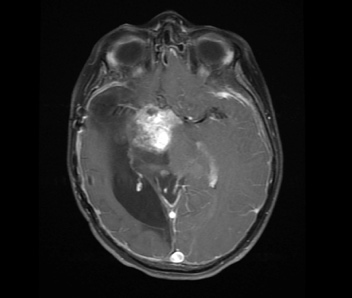 | **Age:** 16  **Sex:** male  **Diagnosis:** pilocytic astrocytoma  **Tumor location:** suprasellar/optic pathway  **Baseline total cystic volume (cm^3^):** 8.7  **Number of follow-up images:** 3  **Treatment duration (months):** 8  **Early timepoint from baseline image (months):** 6  **Late timepoint from baseline image (months):** N/A  **2D measurement:** T1CE coronal  **Solid volume measurement:** T1CE |
| PNOC001-40 | 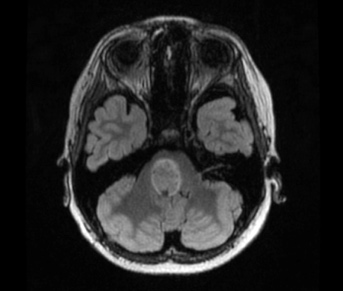 | **Age:** 8  **Sex:** male  **Diagnosis:** astrocytoma  **Tumor location:** posterior fossa  **Baseline total cystic volume (cm^3^):** 1.9 (cyst appears on 2nd follow-up)  **Number of follow-up images:** 2  **Treatment duration (months):** 3  **Early timepoint from baseline image (months):** 3  **Late timepoint from baseline image (months):** N/A  **2D measurement:** FLAIR axial  **Solid volume measurement:** FLAIR minus total cyst |
| PNOC001-41 | 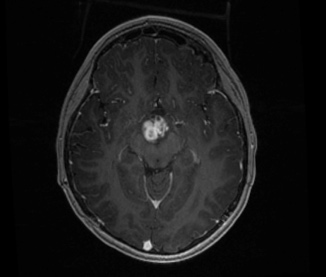 | **Age:** 11  **Sex:** female  **Diagnosis:** pilocytic astrocytoma  **Tumor location:** suprasellar/optic pathway  **Baseline total cystic volume (cm^3^):** 3.4  **Number of follow-up images:** 10  **Treatment duration (months):** 22  **Early timepoint from baseline image (months):** 6  **Late timepoint from baseline image (months):** 23  **2D measurement:** T1CE coronal  **Solid volume measurement:** T1CE |
| PNOC001-42 | 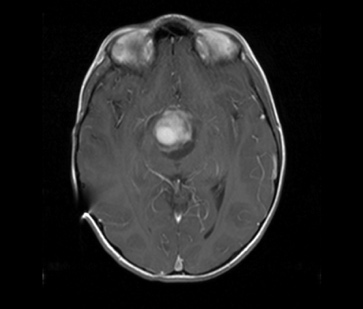 | **Age:** 10  **Sex:** male  **Diagnosis:** astrocytoma  **Tumor location:** suprasellar/optic pathway  **Baseline total cystic volume (cm^3^):** 8.8  **Number of follow-up images:** 2  **Treatment duration (months):** 4  **Early timepoint from baseline image (months):** 4  **Late timepoint from baseline image (months):** N/A  **2D measurement:** T1CE coronal  **Solid volume measurement:** T1CE |
| PNOC001-43 | 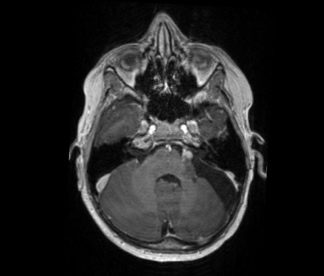 | **Age:** 9  **Sex:** male  **Diagnosis:** pilocytic astrocytoma  **Tumor location:** posterior fossa  **Baseline total cystic volume (cm^3^):** 13.8  **Number of follow-up images:** 3  **Treatment duration (months):** 6  **Early timepoint from baseline image (months):** 6  **Late timepoint from baseline image (months):** N/A  **2D measurement:** T1CE coronal  **Solid volume measurement:** T1CE |
| PNOC001-44 | 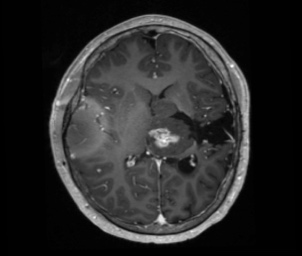 | **Age:** 16  **Sex:** male  **Diagnosis:** astrocytoma  **Tumor location:** supratentorial  **Baseline total cystic volume (cm^3^):** 1.8  **Number of follow-up images:** 3  **Treatment duration (months):** 6  **Early timepoint from baseline image (months):** 6  **Late timepoint from baseline image (months):** N/A  **2D measurement:** T1CE coronal  **Solid volume measurement:** FLAIR minus total cyst |
| PNOC001-45 | 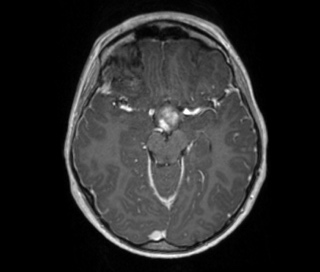 | **Age:** 8  **Sex:** male  **Diagnosis:** pilocytic astrocytoma  **Tumor location:** suprasellar/optic pathway  **Baseline total cystic volume (cm^3^):** 1.6  **Number of follow-up images:** 8  **Treatment duration (months):** 17  **Early timepoint from baseline image (months):** 6  **Late timepoint from baseline image (months):** 17  **2D measurement:** T1CE coronal  **Solid volume measurement:** T1CE |
| PNOC001-47 | 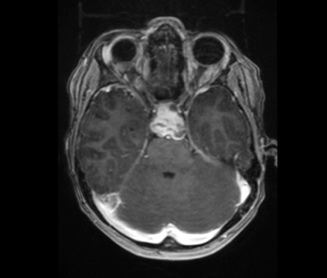 | **Age:** 11  **Sex:** male  **Diagnosis:** pilocytic astrocytoma  **Tumor location:** suprasellar/optic pathway  **Baseline total cystic volume (cm^3^):** 0.8 (cyst appears on first follow-up)  **Number of follow-up images:** 6  **Treatment duration (months):** 11  **Early timepoint from baseline image (months):** 6  **Late timepoint from baseline image (months):** 12  **2D measurement:** T1CE sagittal  **Solid volume measurement:** FLAIR minus total cyst |
| PNOC001-48 | 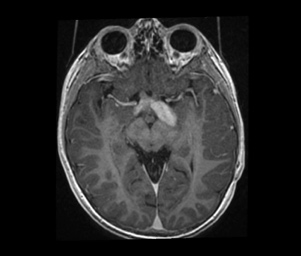 | **Age:** 9  **Sex:** male  **Diagnosis:** pilomyxoid astrocytoma  **Tumor location:** suprasellar/optic pathway  **Baseline total cystic volume (cm^3^):** 0.4  **Number of follow-up images:** 9  **Treatment duration (months):** 22  **Early timepoint from baseline image (months):** 6  **Late timepoint from baseline image (months):** 23  **2D measurement:** T1CE axial  **Solid volume measurement:** FLAIR minus total cyst |
| PNOC001-50 | 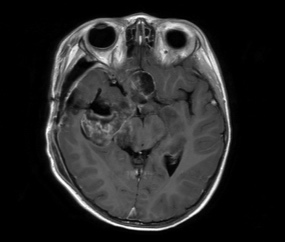 | **Age:** 5  **Sex:** female  **Diagnosis:** pilocytic astrocytoma  **Tumor location:** suprasellar/optic pathway  **Baseline total cystic volume (cm^3^):** 8  **Number of follow-up images:** 4  **Treatment duration (months):** 5  **Early timepoint from baseline image (months):** 6  **Late timepoint from baseline image (months):** N/A  **2D measurement:** T1CE axial  **Solid volume measurement:** FLAIR minus total cyst |
| PNOC001-52 | 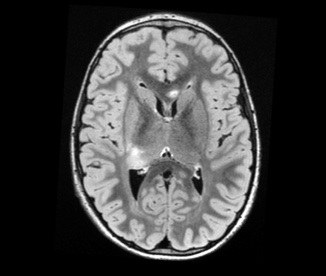 | **Age:** 8  **Sex:** male  **Diagnosis:** astrocytoma  **Tumor location:** supratentorial  **Baseline total cystic volume (cm^3^):** N/A  **Number of follow-up images:** 7  **Treatment duration (months):** 11  **Early timepoint from baseline image (months):** 6  **Late timepoint from baseline image (months):** 13  **2D measurement:** FLAIR axial  **Solid volume measurement:** FLAIR |
| PNOC001-53 | 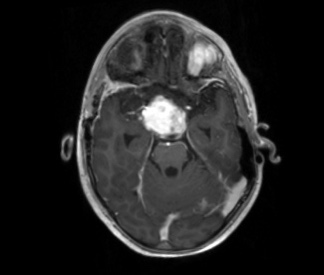 | **Age:** 6  **Sex:** male  **Diagnosis:** pilocytic astrocytoma  **Tumor location:** posterior fossa  **Baseline total cystic volume (cm^3^):** 15.1  **Number of follow-up images:** 3  **Treatment duration (months):** 7  **Early timepoint from baseline image (months):** 4  **Late timepoint from baseline image (months):** N/A  **2D measurement:** T1CE sagittal  **Solid volume measurement:** T1CE |
| PNOC001-55 | 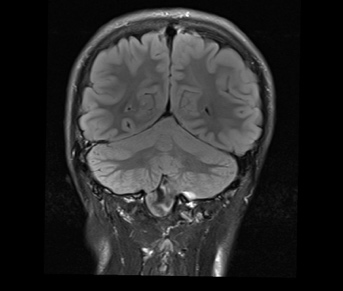 | **Age:** 13  **Sex:** male  **Diagnosis:** pilocytic astrocytoma  **Tumor location:** posterior fossa  **Baseline total cystic volume (cm^3^):** N/A  **Number of follow-up images:** 6  **Treatment duration (months):** 11  **Early timepoint from baseline image (months):** 6  **Late timepoint from baseline image (months):** 12  **2D measurement:** FLAIR coronal  **Solid volume measurement:** FLAIR |
| PNOC001-56 | 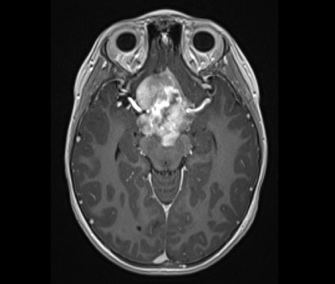 | **Age:** 4  **Sex:** female  **Diagnosis:** pilomyxoid astrocytoma  **Tumor location:** suprasellar/optic pathway  **Baseline total cystic volume (cm^3^):** 0.2  **Number of follow-up images:** 1  **Treatment duration (months):** 2  **Early timepoint from baseline image (months):** 2  **Late timepoint from baseline image (months):** N/A  **2D measurement:** T1CE sagittal  **Solid volume measurement:** T1CE |

| PNOC001-57 | 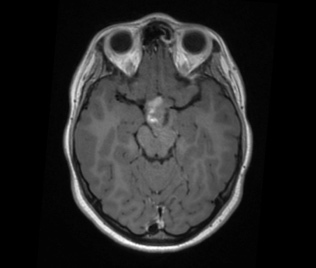 | **Age:** 16  **Sex:** female  **Diagnosis:** pilocytic astrocytoma  **Tumor location:** suprasellar/optic pathway  **Baseline total cystic volume (cm^3^):** 0.3  **Number of follow-up images:** 7  **Treatment duration (months):** 22  **Early timepoint from baseline image (months):** 6  **Late timepoint from baseline image (months):** 17  **2D measurement:** T1CE sagittal  **Solid volume measurement:** FLAIR minus total cyst |
| --- | --- | --- |
| PNOC001-58 | 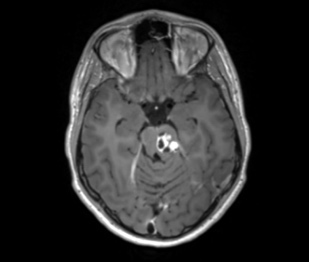 | **Age:** 18  **Sex:** male  **Diagnosis:** pilocytic astrocytoma  **Tumor location:** posterior fossa  **Baseline total cystic volume (cm^3^):** 1.7  **Number of follow-up images:** 5  **Treatment duration (months):** 22  **Early timepoint from baseline image (months):** 6  **Late timepoint from baseline image (months):** 10  **2D measurement:** T1CE axial  **Solid volume measurement:** T1CE minus cyst within enhancing volume |
| PNOC001-59 | 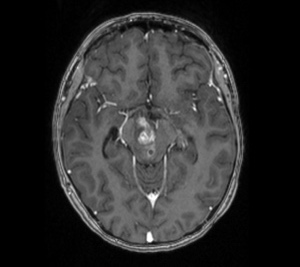 | **Age:** 14  **Sex:** female  **Diagnosis:** pilocytic astrocytoma  **Tumor location:** posterior fossa  **Baseline total cystic volume (cm^3^):** 0.3  **Number of follow-up images:** 8  **Treatment duration (months):** 22  **Early timepoint from baseline image (months):** 6  **Late timepoint from baseline image (months):** 17  **2D measurement:** T1CE axial  **Solid volume measurement:** T1CE |
| PNOC001-61 | 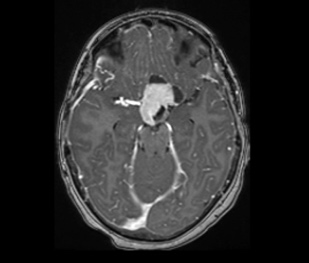 | **Age:** 6  **Sex:** female  **Diagnosis:** pilomyxoid astrocytoma  **Tumor location:** suprasellar/optic pathway  **Baseline total cystic volume (cm^3^):** 7.2  **Number of follow-up images:** 3  **Treatment duration (months):** 11  **Early timepoint from baseline image (months):** 6  **Late timepoint from baseline image (months):** N/A  **2D measurement:** T1CE sagittal  **Solid volume measurement:** T1CE |
| PNOC001-62 | 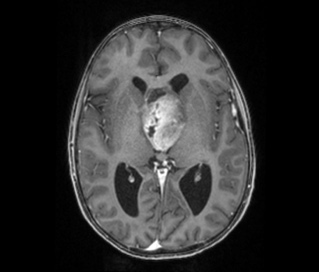 | **Age:** 11  **Sex:** male  **Diagnosis:** pilocytic astrocytoma  **Tumor location:** suprasellar/optic pathway  **Baseline total cystic volume (cm^3^):** 4.8  **Number of follow-up images:** 6  **Treatment duration (months):** 24  **Early timepoint from baseline image (months):** 6  **Late timepoint from baseline image (months):** 12  **2D measurement:** T1CE sagittal  **Solid volume measurement:** T1CE |
| PNOC001-63 | 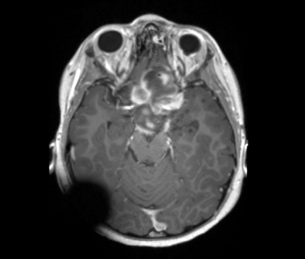 | **Age:** 9  **Sex:** female  **Diagnosis:** pilocytic astrocytoma  **Tumor location:** suprasellar/optic pathway  **Baseline total cystic volume (cm^3^):** 17.3  **Number of follow-up images:** 2  **Treatment duration (months):** 4  **Early timepoint from baseline image (months):** 4  **Late timepoint from baseline image (months):** N/A  **2D measurement:** T1CE sagittal  **Solid volume measurement:** T1CE minus cyst within enhancing volume |
| PNOC001-65 | 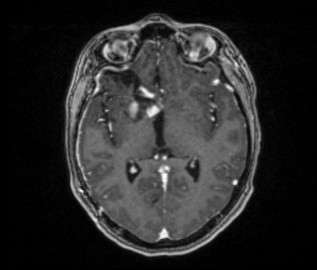 | **Age:** 15  **Sex:** female  **Diagnosis:** glioneuronal tumor  **Tumor location:** supratentorial  **Baseline total cystic volume (cm^3^):** 1.7  **Number of follow-up images:** 3  **Treatment duration (months):** 6.5  **Early timepoint from baseline image (months):** 7  **Late timepoint from baseline image (months):** N/A  **2D measurement:** T1CE axial  **Solid volume measurement:** T1CE |
| PNOC001-66 | 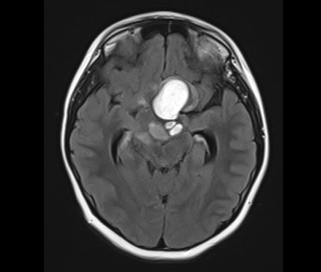 | **Age:** 5  **Sex:** female  **Diagnosis:** pilomyxoid astrocytoma  **Tumor location:** suprasellar/optic pathway  **Baseline total cystic volume (cm^3^):** 5.8  **Number of follow-up images:** 5  **Treatment duration (months):** 17  **Early timepoint from baseline image (months):** 6  **Late timepoint from baseline image (months):** 12  **2D measurement:** T2 axial  **Solid volume measurement:** FLAIR minus total cyst |

**Table S1.** Baseline images and participant details.


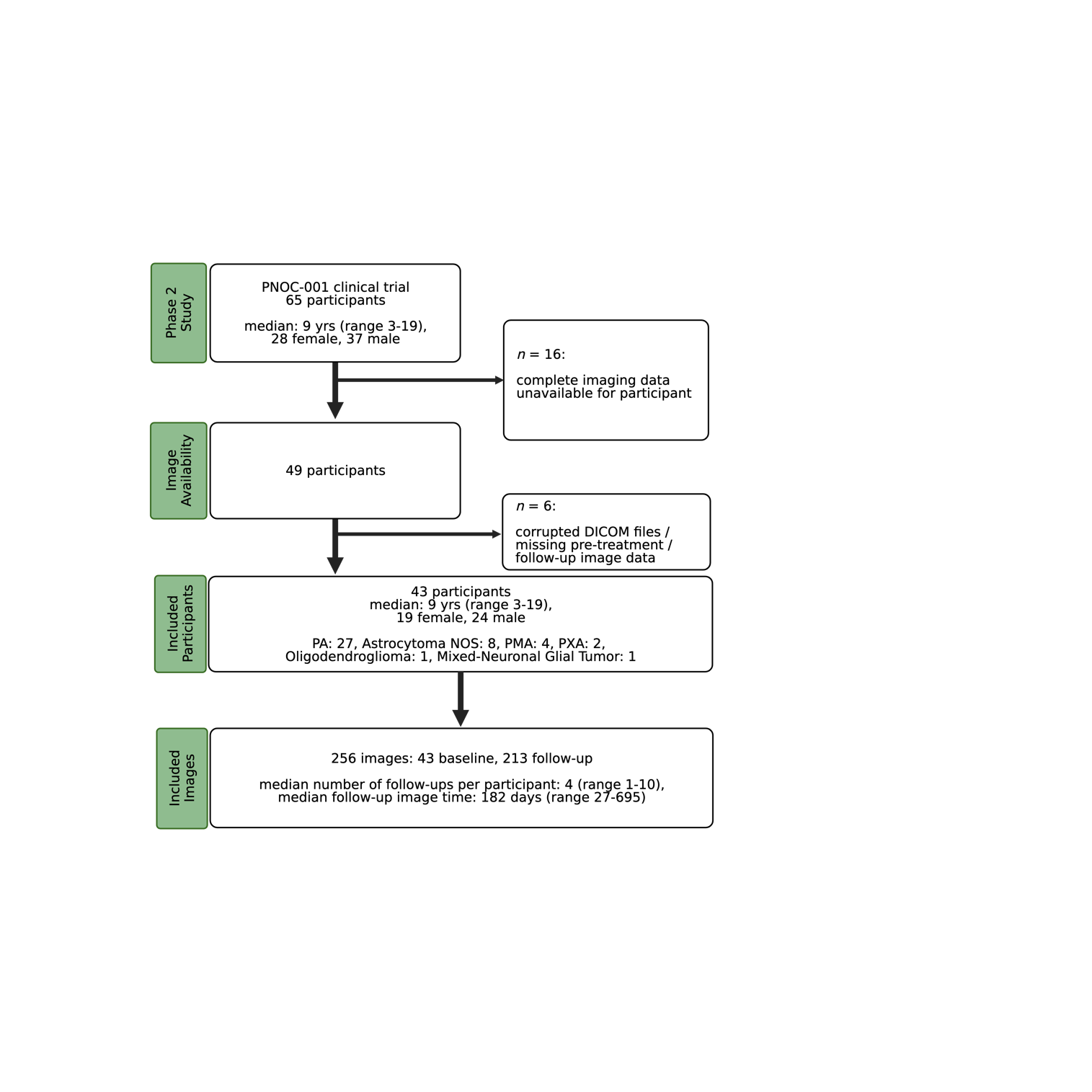


Created with BioRender.com

**Figure S2.** After exclusion of participants with missing imaging data or corrupted DICOM files, 43 participants were included for volumetric analysis.


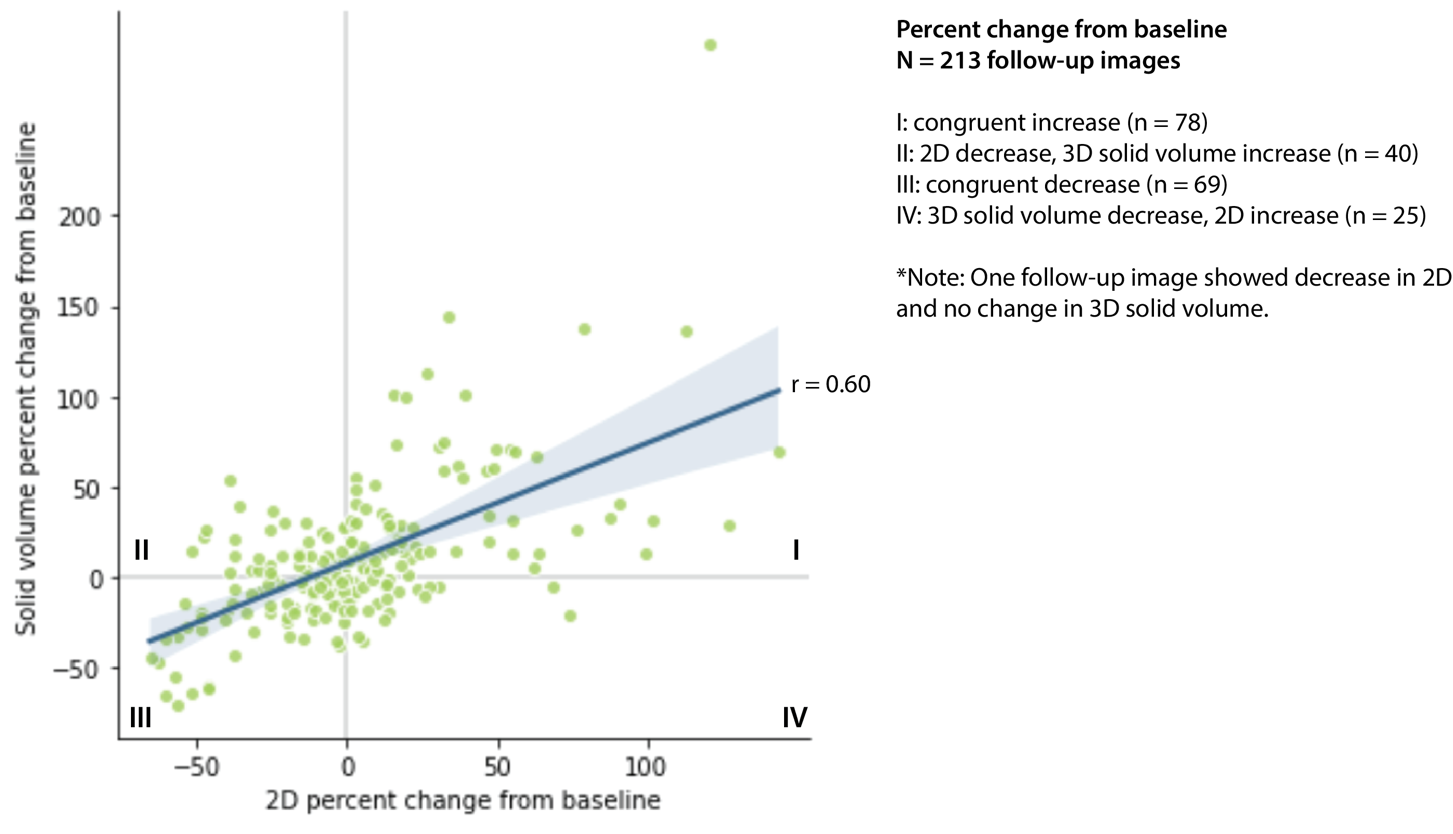


**Figure S3.** Correlation between 2D and 3D solid volume percent change for all follow-up images (n = 213).

3 Tumor Growth Model

**3.1 Model definition**

Following the work of Glazar *et al.* and Brueningk *et al.* [[15](#_bookmark16), [16](#_bookmark17)] model tumor volume *V* is represented as growing exponentially in the absence of treatment. Given the limited number of pre-treatment data points we consider this a reasonable trade-off between the required model complexity to allow for flexibility to fit all included patients, and the underlying biological growth behavior.


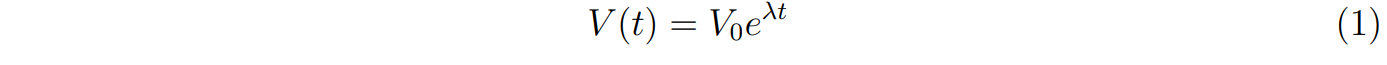


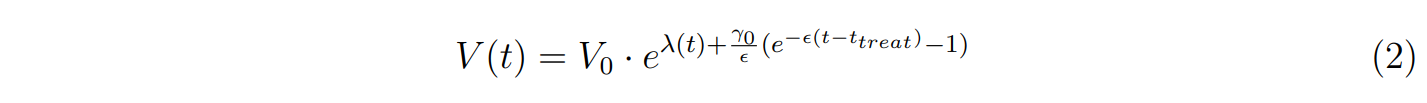
Here, *λ* is the growth rate, and *V*_0_ the tumor volume at the first measurement time point (*t*_0_ = 0). Treatment (initiated at time *t_treat_*), is described as the counterplay of tumor regression in response to therapy and the onset of treatment resistance as previously described:

In this formulation *γ*_0_ describes the initial rate of volume reduction in response to therapy. *ϵ* characterizes the increase of therapy resistance as an exponential decay rate since treatment initiation at time *t_treat_*.

In summary, this model comprises four free parameters, namely the initial tumor volume *V*_0_, the growth rate *λ*, the initial treatment response rate *γ*_0_, and the rate of resistance *ϵ*. Given the highly limited number of tumor volume measurements, we decided to fix the initial tumor volume to the measured data point and further, following previous recommendations [[15](#_bookmark16), [16](#_bookmark17)] assumed a constant growth rate across all patients.

**3.2 Model fitting**

For each participant up to two evaluation endpoints with BT-RADS assessment were considered for independent model fitting. Models were fit to tumor growth curve data using the *lmfit* library (version 1.0.2) in python with fit parameter posterior probability distributions being estimated using the Markov Chain Monte Carlo method implemented as the EMCEE package in the *lmfit* library. 10k steps were simulated; additionally, measurement uncertainty was accounted for by bootstrapping: we repeated the fit procedure n = 200 times for each participant and randomly shift the target volume data based on a normal distribution with a mean corresponding to the true value, and standard deviation reflecting the estimated measurement uncertainty. We used a delineation uncertainty estimate of 10% of baseline tumor volume. This may be a strong simplification, as measurement uncertainty can vary for each participant depending on the specific tumor characteristics in terms of volume, infiltration, and image contrast. For each participant the reported therapy initiation days are used for the parameter t_treat_.

Fit results are reported as mean values and 95% confidence bounds over all bootstraps. Fit performance is scored using coefficients of determination (*R*^2^) between the true and predicted (median) volumes. Fit parameters, and the time to reach the minimum tumor volume *t_V_min* are reported as mean values with standard deviations over all bootstraps. For the final application, some simplifying assumptions were made to balance the trade-off between limited data availability for fitting and model complexity. First, given the absence of more than one pre-treatment data point for the majority of patients in this cohort we fixed the initial tumor volume to the first observed one. Two participants (PNOC001-14 and -62) did not present with a pretreatment scan. Here, the first available image was used for reference. Secondly, we assume a constant growth rate for all participants. The optimal growth rate was identified through grid search covering a clinically realistic range between doubling times of 5 and 3200 days for these participants. We sum all ranks (within the range of assessed growth rates per participant) of coefficients of determination over all participants as shown in **Figure S4**. This yielded an optimal tumor doubling time of 100 days corresponding to a growth rate of 0.0069/day.


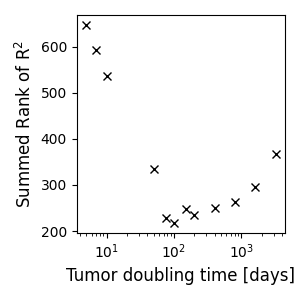


**Figure S4.** Evaluation of the optimal growth rate used uniformly for all participants. The lowest summed rank of *R*^2^ was found for a doubling time of 100 days.

**3.2 Participant tumor growth curves**


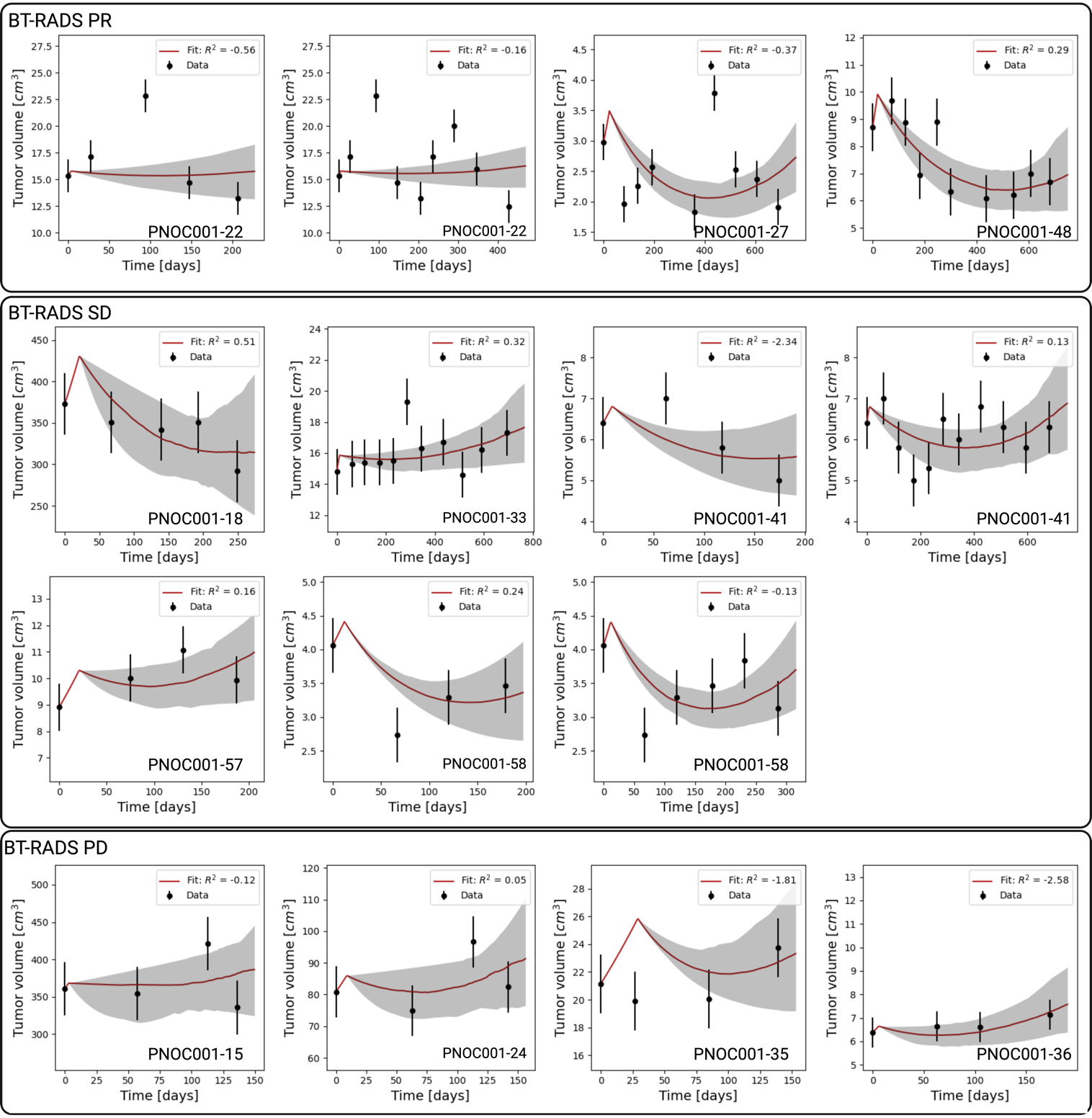


**Figure S5.** Overview over all tumor growth curves yielding different classification by BT- RADS and model-based label assignment as indicated by subset headings. The model takes the overall data trend into account but may also be driven by the estimated uncertainty range. Note that a participant is included with up to two (early and late) BT-RADS assessment endpoints resulting in a different number of scans included for model fitting.

**
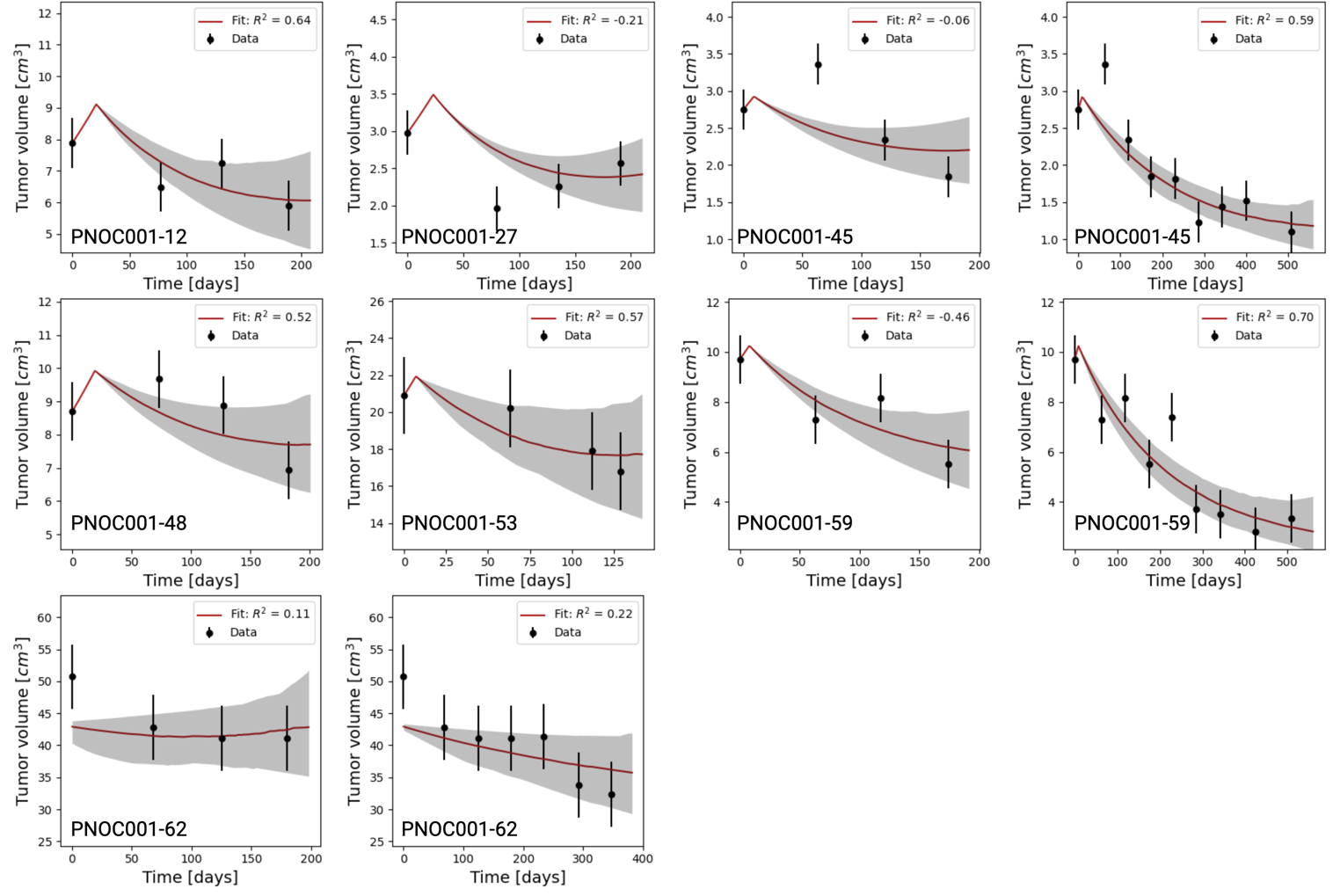
**

**Figure S6.** Overview over all tumor growth curves assigned as PR in both BT-RADS and model-based label assignment. Note that a participant is included with up to two (early and late) BT-RADS assessment endpoints resulting in a different number of scans included for model fitting.


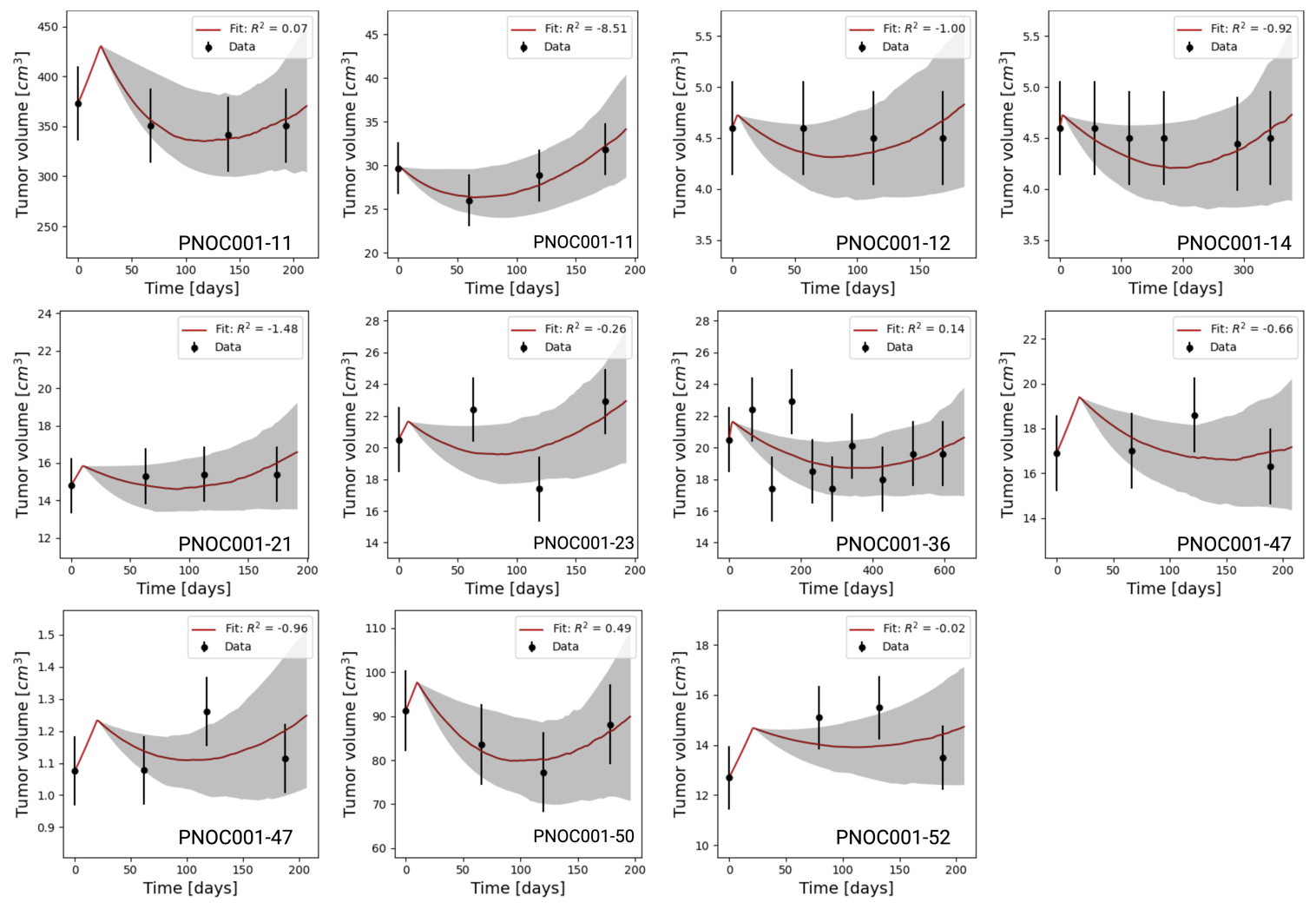


**Figure S7.** Overview over all tumor growth curves assigned as SD in both BT-RADS and model-based label assignment. Note that a participant is included with up to two (early and late) BT-RADS assessment endpoints resulting in a different number of scans included for model fitting.

**
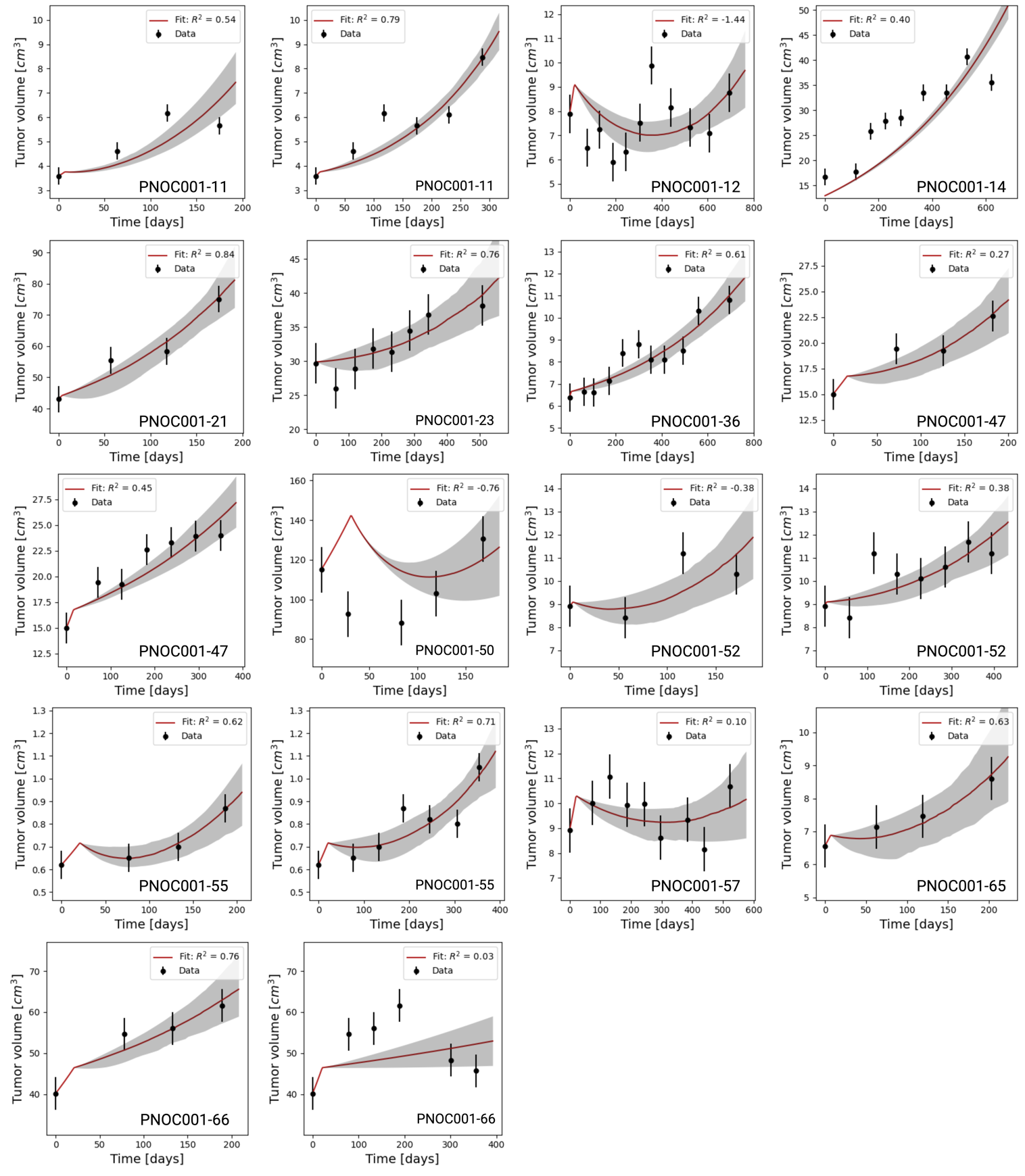
**

**Figure S8.** Overview over all tumor growth curves assigned as PD in both BT-RADS and model-based label assignment. Note that a participant is included with up to two (early and late) BT-RADS assessment endpoints resulting in a different number of scans included for model fitting.

|  | **γ0 x 10^3^** | **ε** | **t_Vmin_ [Days]** |
| --- | --- | --- | --- |
| **11_EARLY** | 7.51 (5.17, 8.44) | 0.01045 (0.00461, 0.01871) | 14.1 (-56.1, 24.2) |
| **11_LATE** | 5.64 (4.46, 7.44) | 0.00250 (0.00089, 0.00533) | -73.1 (-408.1, 20.4) |
| **12_EARLY** | 11.92 (9.63, 13.47) | 0.00299 (0.00068, 0.00572) | 199.7 (112.2, 612.7) |
| **12_LATE** | 8.53 (7.67, 9.68) | 0.00061 (0.00027, 0.00116) | 358.0 (303.9, 501.7) |
| **14_LATE** | 4.96 (4.87, 5.09) | 0.00001 (0.00001, 0.00002) | -25151.6 (-33587.4, -17183.7) |
| **15_EARLY** | 7.14 (5.90, 13.84) | 0.00003 (0.00000, 0.01712) | 48.1 (-20316.8, 13645.9) |
| **18_EARLY** | 12.52 (7.57, 13.86) | 0.00559 (0.00001, 0.00973) | 128.8 (92.2, 24811.5) |
| **18_LATE** | 10.16 (7.68, 13.86) | 0.00174 (0.00000, 0.00633) | 245.7 (126.5, 108241.9) |
| **21_EARLY** | 4.50 (3.62, 12.47) | 0.00177 (0.00025, 0.02349) | -249.0 (-2438.7, 29.0) |
| **22_EARLY** | 7.46 (6.41, 8.78) | 0.00062 (0.00042, 0.00135) | 115.2 (-98.5, 389.1) |
| **22_LATE** | 7.10 (6.79, 7.51) | 0.00014 (0.00008, 0.00031) | 177.1 (-164.2, 536.5) |
| **23_EARLY** | 11.19 (6.62, 13.40) | 0.00677 (0.00065, 0.01151) | 68.6 (-39.0, 231.2) |
| **23_LATE** | 6.83 (6.26, 7.99) | 0.00026 (0.00004, 0.00105) | -53.8 (-2037.8, 146.9) |
| **24_EARLY** | 9.29 (5.84, 13.71) | 0.00499 (0.00012, 0.01609) | 62.2 (-1064.8, 747.0) |
| **27_EARLY** | 12.47 (11.88, 13.03) | 0.00379 (0.00197, 0.00597) | 177.7 (115.7, 330.4) |
| **27_LATE** | 9.69 (8.72, 10.73) | 0.00081 (0.00047, 0.00121) | 431.3 (361.3, 563.8) |
| **30_EARLY** | 9.34 (6.97, 12.92) | 0.00351 (0.00037, 0.00948) | 84.8 (14.1, 408.6) |
| **30_LATE** | 8.10 (7.20, 9.65) | 0.00079 (0.00019, 0.00225) | 187.2 (82.0, 537.2) |
| **33_EARLY** | 8.84 (6.78, 12.91) | 0.00291 (0.00055, 0.00984) | 85.3 (-20.2, 332.7) |
| **33_LATE** | 7.09 (6.75, 7.70) | 0.00011 (0.00004, 0.00044) | 206.7 (-479.2, 566.3) |
| **35_EARLY** | 12.35 (9.67, 13.16) | 0.00785 (0.00346, 0.01569) | 99.6 (68.1, 197.2) |
| **36_EARLY** | 9.07 (6.69, 13.50) | 0.00433 (0.00042, 0.01219) | 65.2 (-60.4, 157.3) |
| **36_LATE** | 6.50 (6.30, 7.20) | 0.00014 (0.00004, 0.00047) | -457.3 (-2186.5, 87.7) |
| **37_EARLY** | 9.77 (6.78, 12.69) | 0.00468 (0.00085, 0.01013) | 82.8 (-10.4, 280.5) |
| **37_LATE** | 7.75 (7.26, 8.71) | 0.00032 (0.00006, 0.00080) | 360.8 (242.0, 1208.7) |
| **39_EARLY** | 9.62 (7.67, 12.84) | 0.00246 (0.00020, 0.00707) | 149.8 (91.4, 615.7) |
| **41_EARLY** | 9.95 (8.25, 12.39) | 0.00236 (0.00103, 0.00598) | 161.2 (80.4, 275.0) |
| **41_LATE** | 7.86 (7.34, 9.02) | 0.00034 (0.00013, 0.00080) | 364.1 (277.7, 715.1) |
| **43_EARLY** | 9.69 (7.47, 12.28) | 0.00389 (0.00085, 0.00845) | 105.7 (61.2, 318.8) |
| **44_EARLY** | 11.49 (6.91, 13.80) | 0.00531 (0.00007, 0.01062) | 109.0 (-1.5, 2315.3) |
| **45_EARLY** | 10.68 (9.16, 12.16) | 0.00265 (0.00134, 0.00382) | 175.4 (105.1, 428.0) |
| **45_LATE** | 10.03 (8.97, 11.98) | 0.00055 (0.00015, 0.00154) | 651.8 (363.5, 1694.9) |
| **47_EARLY** | 6.64 (5.16, 10.87) | 0.00299 (0.00039, 0.01185) | 3.5 (-704.6, 57.5) |
| **47_LATE** | 5.89 (5.52, 6.33) | 0.00023 (0.00015, 0.00088) | -720.2 (-1318.6, -86.8) |
| **48_EARLY** | 9.92 (8.48, 11.44) | 0.00197 (0.00049, 0.00463) | 199.7 (116.4, 555.5) |
| **48_LATE** | 8.75 (7.99, 10.07) | 0.00047 (0.00011, 0.00105) | 520.3 (374.0, 1390.8) |
| **50_EARLY** | 13.83 (10.97, 13.84) | 0.00850 (0.00361, 0.01175) | 112.3 (88.4, 162.3) |
| **52_EARLY** | 8.63 (6.06, 11.66) | 0.00505 (0.00164, 0.01239) | 45.1 (-78.6, 87.3) |
| **52_LATE** | 6.80 (6.32, 7.92) | 0.00042 (0.00013, 0.00127) | -45.0 (-469.4, 107.3) |
| **53_EARLY** | 10.59 (8.48, 13.32) | 0.00273 (0.00095, 0.00934) | 145.1 (67.6, 346.4) |
| **55_EARLY** | 11.03 (6.59, 13.32) | 0.00871 (0.00128, 0.01322) | 72.8 (-18.5, 107.6) |
| **55_LATE** | 7.81 (6.37, 10.05) | 0.00176 (0.00034, 0.00366) | 87.1 (-160.5, 128.6) |
| **57_EARLY** | 8.61 (6.48, 11.14) | 0.00281 (0.00026, 0.00881) | 91.9 (-242.9, 194.0) |
| **57_LATE** | 7.73 (7.21, 8.80) | 0.00037 (0.00010, 0.00108) | 301.4 (207.7, 627.4) |
| **58_EARLY** | 12.36 (11.46, 13.33) | 0.00448 (0.00234, 0.00695) | 141.1 (96.9, 250.6) |
| **58_LATE** | 11.55 (9.45, 13.01) | 0.00314 (0.00140, 0.00514) | 179.0 (133.7, 295.3) |
| **59_EARLY** | 12.03 (9.87, 13.43) | 0.00226 (0.00048, 0.00514) | 247.9 (127.4, 838.7) |
| **59_LATE** | 10.85 (9.60, 12.30) | 0.00057 (0.00020, 0.00157) | 792.3 (367.5, 2151.6) |
| **61_EARLY** | 8.13 (6.71, 10.85) | 0.00167 (0.00061, 0.00599) | 109.5 (-10.1, 240.4) |
| **62_EARLY** | 7.62 (6.46, 12.96) | 0.00038 (0.00010, 0.00822) | 110.4 (-267.9, 1526.2) |
| **62_LATE** | 7.59 (6.94, 8.46) | 0.00006 (0.00002, 0.00073) | 1329.1 (-6.5, 4608.4) |
| **65_EARLY** | 7.77 (6.03, 12.77) | 0.00311 (0.00047, 0.01159) | 41.2 (-280.2, 84.5) |
| **66_EARLY** | 5.50 (4.73, 8.72) | 0.00061 (0.00017, 0.00827) | -392.9 (-1930.3, 53.4) |

**Table S2.** Overview of the model fit parameters γ_0_ and ε, as well as the time to best response t_Vmin_. All values are given as median values and full ranges over all bootstraps.

References

[1] Ryall S, Zapotocky M, Fukuoka K, et al. Integrated Molecular and Clinical Analysis of 1,000 Pediatric Low-Grade Gliomas. *Cancer Cell*. 2020;37(4):569-583.e5.

[2] Guertin DA, Sabatini DM. Defining the role of mTOR in cancer. *Cancer Cell*. 2007;12(1):9-22.

[3] Colardo M, Segatto M, Di Bartolomeo S. Targeting RTK-PI3K-mTOR Axis in Gliomas: An Update. *Int J Mol Sci*. 2021;22(9):4899.

[4] Mendoza MC, Er EE, Blenis J. The Ras-ERK and PI3K-mTOR pathways: cross-talk and compensation. *Trends Biochem Sci*. 2011;36(6):320-328.

[5] Wright KD, Yao X, London WB, et al. A POETIC Phase II study of continuous oral everolimus in recurrent, radiographically progressive pediatric low-grade glioma. *Pediatr Blood Cancer*. 2021;68(2):e28787.

[6] Atkins MB, Yasothan U, Kirkpatrick P. Everolimus. *Nat Rev Drug Discov*. 2009;8(7):535-536.

[7] Mueller S, Aboian M, Nazemi K, et al. LGG-53. PNOC001 (NCT01734512): A PHASE II STUDY OF EVEROLIMUS FOR RECURRENT OR PROGRESSIVE PEDIATRIC LOW-GRADE GLIOMAS (pLGG). *Neuro Oncol*. 2020;22(Suppl 3):iii376.

[8] Ullrich NJ, Prabhu SP, Reddy AT, et al. A phase II study of continuous oral mTOR inhibitor everolimus for recurrent, radiographic-progressive neurofibromatosis type 1-associated pediatric low-grade glioma: a Neurofibromatosis Clinical Trials Consortium study. *Neuro Oncol*. 2020;22(10):1527-1535.

[9] Wen PY, Macdonald DR, Reardon DA, et al. Updated response assessment criteria for high-grade gliomas: response assessment in neuro-oncology working group. *J Clin Oncol*. 2010;28(11):1963-1972.

[10] Ellingson BM, Wen PY, Cloughesy TF. Modified Criteria for Radiographic Response Assessment in Glioblastoma Clinical Trials. *Neurotherapeutics*. 2017;14(2):307-320.

[11] Macdonald DR, Cascino TL, Schold SC Jr, Cairncross JG. Response criteria for phase II studies of supratentorial malignant glioma. *J Clin Oncol*. 1990;8(7):1277-1280.

[12] Therasse, P., Arbuck, S.G., Eisenhauer, E.A. *et al.* New guidelines to evaluate the response to treatment in solid tumors. *Breast Cancer.* 2005;12(Suppl 1): S16–S27.

[13] Warren KE, Poussaint TY, Vezina G, et al. Challenges with defining response to antitumor agents in pediatric neuro-oncology: a report from the response assessment in pediatric neuro-oncology (RAPNO) working group. *Pediatr Blood Cancer*. 2013;60(9):1397-1401.

[14] Fangusaro J, Bandopadhayay P. The “Risk” in Pediatric Low-Grade Glioma. *Cancer Cell*. 2020;37(4):424–425.

[15] Glazar DJ, Grass GD, Arrington JA, et al. Tumor Volume Dynamics as an Early Biomarker for Patient-Specific Evolution of Resistance and Progression in Recurrent High-Grade Glioma. *J Clin Med*. 2020;9(7):2019.

[16] Bru¨ningk SC, Peacock J, Whelan CJ, et al. Intermittent radiotherapy as alternative treatment for recurrent high grade glioma: a modeling study based on longitudinal tumor measurements. *Scientific Reports*. 2021;11(1): 20219.
